# Exploring Artificial Intelligence in Orthopedic Surgery: A Review of Perception, Decision, and Execution Systems

**DOI:** 10.3390/s26092591

**Published:** 2026-04-22

**Authors:** Dehan Li, Wanshi Liu, Md. Mihraz Hossain Niloy, Zhang Yi, Lei Xu

**Affiliations:** Machine Intelligence Laboratory, College of Computer Science, Sichuan University, No. 24 South Section 1, Yihuan Road, Chengdu 610065, China; hooraylee@stu.scu.edu.cn (D.L.); liuwanshi@stu.scu.edu.cn (W.L.); mihrazhossain@stu.scu.edu.cn (M.M.H.N.); zhangyi@scu.edu.cn (Z.Y.)

**Keywords:** artificial intelligence, orthopedic surgery, surgical navigation, machine learning, deep learning

## Abstract

Artificial intelligence (AI) has become an indispensable tool in orthopedic surgery. It provides new methods to increase surgical precision, improve patient safety, and support personalized treatment plans. This review presents a comprehensive analysis of AI-assisted orthopedic surgery across three core domains. Based on 89 recent studies, this review organizes findings around a perception–decision–execution framework. It groups diverse AI applications into certain categories while highlighting the mutuality across domains. Perception systems have progressed from basic CNN-based segmentation models to advanced transformer architectures. They support multi-modal data fusion and enable uncertainty quantification. Decision systems have moved far beyond rigid rule-based methods and evolve into data-driven models that support surgical planning, accurate risk prediction and continuous outcome optimization. And execution systems have advanced from passive navigation tools to active robotic assistance systems with real-time adaptive capabilities. Beyond mapping technological advances, this review also identifies pivotal challenges that hinder clinical translation and concludes with a clear roadmap for future research, which marks closed-loop surgical assistance systems as the next key development direction. Building on these findings, this review illuminates the potential of AI-assisted orthopedic surgery and guides future research toward innovations that can be translated into clinical practice.

## 1. Introduction

Orthopedic surgery has reached a pivotal crossroads. On the one hand, clinical demand for precision, personalized care, and improved patient outcomes is steadily rising. On the other hand, the field must contend with the innate intricacy of musculoskeletal anatomy and the limitations of traditional surgical practices. Each year, millions of individuals undergo orthopedic interventions, such as joint replacement and spinal surgery. However, Heydar et al. [1], Zhang et al. [2] highlighted that many of these patients face risks such as complications, suboptimal outcomes, and prolonged recovery times. Surgeons face these challenges as they must make complex decisions with limited visual and tactile feedback. They often work in confined spaces where the margin for error is small. At the same time, the global population is aging, which leads to a higher prevalence of musculoskeletal disorders. Consequently, the demand for more effective surgical solutions is now greater than ever.

Artificial intelligence (AI) has recently become a powerful tool in healthcare. It offers new ways to solve the difficult problems found in orthopedic surgery. Technologies such as machine learning, computer vision, and robotics show great potential to make surgery more precise and keep patients safer. These tools also help doctors create treatment plans tailored to individual patients. Early landmark studies established the basis for this progress. For example, Kuok et al. [3] focused on anatomical structure identification, and Zhang et al. [4] addressed preoperative path planning. Such early successes started a wave of new research, with hundreds of studies exploring AI applications across various orthopedic procedures.

**Figure 1:**
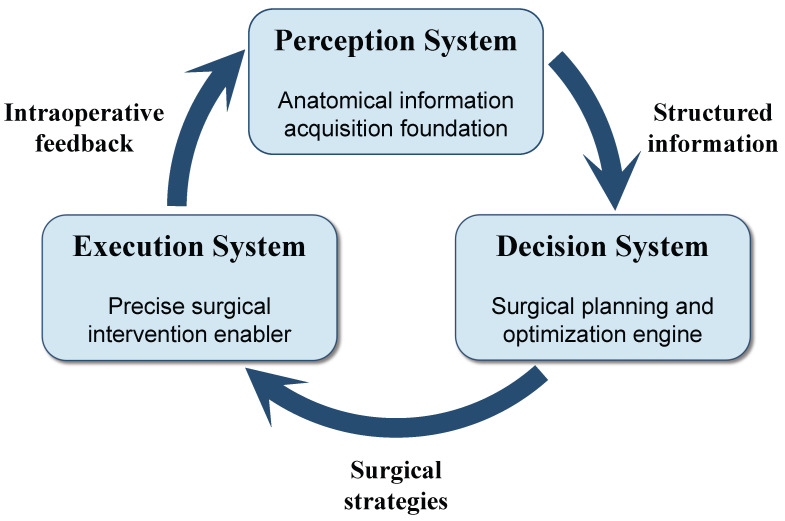
Core components of integrated AI system in orthopedic surgery.

Today, AI-assisted orthopedic surgery has moved beyond isolated technical innovations. It now uses integrated systems that support the entire surgical workflow. These systems consist of three main parts that work together. Figure 1 illustrates these core interconnected components. Perception systems use computer vision to segment anatomical structures, detect landmarks, and track surgical instruments. Decision systems apply machine learning to predict outcomes and optimize treatment plans. These systems also offer clinical decision support. Execution systems combine robotic control and augmented reality to enable precise interventions. This change represents a broader shift in AI research. The focus has moved away from just developing individual algorithms. It is now more about building end-to-end systems that can actually tackle those complex real-world problems.

However, the field still faces major challenges. Most current AI models are trained on limited datasets, making them lack generalizability across different anatomical sites, imaging modalities, and patient populations. At the same time, poor interpretability remains a significant challenge for many models. These systems often function as black boxes. The lack of transparency makes it difficult for clinicians to understand the logic behind models. As a result, surgeons may find it hard to trust these tools in a clinical setting. Moreover, existing literature, such as the studies by Pradipta et al. [5], Fan et al. [6], Hu et al. [7], Koryciński et al. [8], often focuses on specific technical tasks. Few studies examine how perception, decision, and execution systems work together, which makes the current view of the field remain fragmented. These obstacles prevent AI research from being widely used in clinical practice.

A critical gap in the current literature is the lack of a comprehensive, systematic review that synthesizes AI applications across the entire orthopedic surgery workflow. Specifically, prior reviews struggle to address two key challenges. First, there is a severe fragmentation in the existing literature; while reviews by Ahmed et al. [9], Li et al. [10], Seetohul et al. [11] focus on narrow technical domains, they fail to address the complex interdependencies and potential integration conflicts that arise when isolated perception, decision, and execution modules are combined into a single workflow. Second, there is a scarcity of reviews specifically tailored to orthopedic surgery, which possesses unique anatomical complexities (e.g., highly articulated joints and rigid bone structures) and surgical requirements distinct from general surgery. This dual gap leaves researchers and clinicians without a unified framework to understand the current state of the art or to identify future research directions.

This study directly addresses these unresolved issues by providing a comprehensive analysis of 89 recent studies on AI-assisted orthopedic surgery. It follows a perception–decision–execution framework and reflects the natural flow of information in intelligent surgical systems. This framework organizes diverse AI applications into coherent categories and explicitly highlights the interdependencies and resolves the integration conflicts among different system components.

This review makes several key contributions to the field of AI-assisted orthopedic surgery. First, this review provides a structured overview of the current state of the art. It covers a wide range of AI applications across the entire surgical workflow. Second, this review identifies critical challenges and limitations. These issues must be resolved before AI research can be used in clinical practice. Third, this review proposes a unified framework to understand AI-assisted orthopedic surgery. This framework emphasizes how perception, decision, and execution systems depend on each other. Finally, this review outlines a clear roadmap for future research, which identifies key directions that will likely drive the field forward in the coming years. This review synthesizes the latest research and offers a comprehensive view of the field. It aims to serve as a valuable resource for researchers, clinicians, and engineers working in AI-assisted orthopedic surgery.

The remaining sections of this review offer a detailed analysis of AI-assisted orthopedic surgery. Section 2 describes the methods used to retrieve the literature. In Section 3, we introduce the key AI models and architectures used in this field. Section 4 examines perception systems that form the anatomical foundation. Section 5 explores decision systems, which transform anatomical information into clinically executable plans. Following this, Section 6 investigates the execution systems that turn those plans into precise surgical interventions. Section 7 addresses common challenges, such as data scarcity and regulatory issues. Finally, Section 8 outlines future research directions, while Section 9 summarizes our key findings of the review.

## 2. Methods

To ensure a rigorous and reproducible methodology, this study was conducted in accordance with the Preferred Reporting Items for Systematic Reviews and Meta-Analyses (PRISMA) guidelines proposed by Page et al. [12]. To identify peer-reviewed studies exploring how artificial intelligence integrates with orthopedic surgical procedures, we conducted a systematic literature search across four major academic databases: IEEE Xplore (https://ieeexplore.ieee.org/, accessed on 19 April 2026), PubMed (https://pubmed.ncbi.nlm.nih.gov/, accessed on 19 April 2026), Web of Science (https://www.webofscience.com/, accessed on 19 April 2026), and Embase (https://www.embase.com/, accessed on 19 April 2026). To ensure comprehensive and accurate coverage of this interdisciplinary field, we designed a retrieval framework using two complementary search strategies. Based on the framework, we constructed the queries to capture both core research themes and specialized domains. This multi-database approach follows established standards in AI-assisted surgical literature to maximize initial recall and ensure that no significant technological breakthroughs were omitted.

### 2.1. Search Query Design

Two tiers of search queries were designed to retrieve relevant literature. To avoid missing studies due to the highly diverse terminology used for specific orthopedic procedures, we intentionally omitted the specific keywords to maximize initial recall, relying instead on strict manual screening to ensure relevance. Thus, a topic-centric retrieval approach was used, covering titles, abstracts, author-defined keywords, and database-assigned metadata. The queries also included appropriate truncation symbols and synonym expansion to maximize recall while maintaining acceptable precision. The specific formulation of the search queries is detailed as follows:

1. Primary Core Query: This query was designed to identify the fundamental intersection of minimally invasive surgery and general AI methods, with the syntax defined as: (surgery OR surgical) AND (minimal* invasi* OR mini-invasive OR minimally-invasive) AND (artificial intelligence OR machine learning OR deep learning OR AI OR DL OR neural network* OR LLM OR VLM OR MLLM OR MVLM OR LVLM OR large language model* OR vision language model*)
2. Expanded Supplementary Query: To broaden the retrieval scope, this query incorporated extended surgical terminology and specialized AI methods, formulated as: (surger* OR surgical OR microsurgery OR surgical procedures OR surgical operations) AND (AI OR AI-* OR CNNs OR Convolutional Neural Network* OR artificial intelligence OR Large Model OR *LLM OR large language model OR deep learning OR multi-mode OR multimode OR multimodal* OR machine learning OR Segment Anything Model OR SAM OR RAG OR retrieval-augmented generation OR DRL OR deep reinforcement learning)

### 2.2. Retrieval Scope and Parameters

We set specific parameters for our literature search to ensure consistency and relevance:

- Time Span: We limited publications to the period from January 2015 to December 2025. This time span allows us to include both established foundational research and the newest advances in the field, showing how the research has evolved over time.
- Language Restriction: Only peer-reviewed publications written in English were included in the initial retrieval pool. This constraint was implemented to eliminate potential biases arising from language barriers and to ensure uniform comprehension and standardized analysis of study content.

### 2.3. Literature Screening Procedure

After gathering initial results from the three databases, we pooled all records generated by both the primary and supplementary queries across the three databases. Duplicate records resulting from overlapping queries and multiple database indexings were then systematically removed using professional bibliographic management software. Then we screened the remaining unique records through a two-step process: first reviewing titles and abstracts, then examining full texts. To minimize selection bias, this screening process was conducted independently by two reviewers (D.L. and W.L.). Any discrepancies regarding study inclusion were resolved through discussion and consensus with a third senior reviewer (L.X.). Throughout this selection, we applied predefined inclusion and exclusion criteria to ensure the final studies were relevant, methodologically sound, and of high quality.

We established our inclusion criteria as follows:

1. Publication Type: The scope was limited to full-text original research articles and full-length peer-reviewed conference proceedings, excluding reviews, editorials, case reports, and conference abstracts. Together, original research articles and high-quality conference proceedings provide comprehensive literature support, capturing both in-depth systematic evaluations and cutting-edge algorithmic breakthroughs. This ensures we have access to detailed methodology and results data.
2. Methodology: Studies needed to have deep learning or machine learning algorithms as their central methodological component.
3. Application Domain: The algorithms had to be specifically developed, tested, or used in orthopedic surgical procedures, such as joint replacement and spinal surgery. This criterion served as the primary filter to extract orthopedic-specific literature from the broadly retrieved records.
4. Functional Classification: The algorithms had to fit into at least one of three functional categories: (1) Perception systems like medical image segmentation and surgical instrument tracking; (2) Decision-making systems including surgical outcome prediction and personalized surgical planning; (3) Execution systems, involving functions such as surgical robot control and real-time motion guidance.

**Figure 2:**
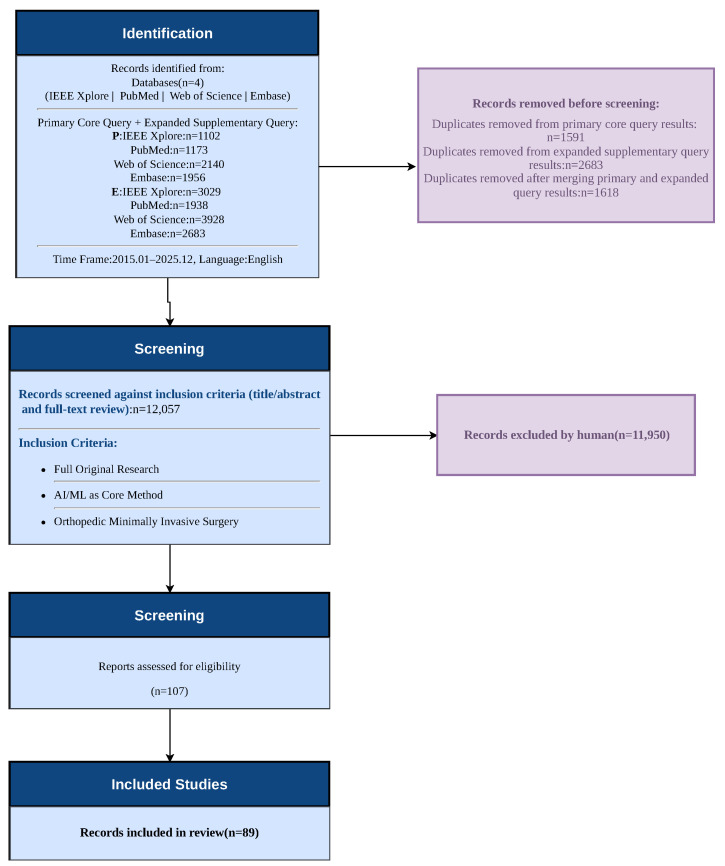
Flowchart of the systematic literature search and screening process.

**Figure 3:**
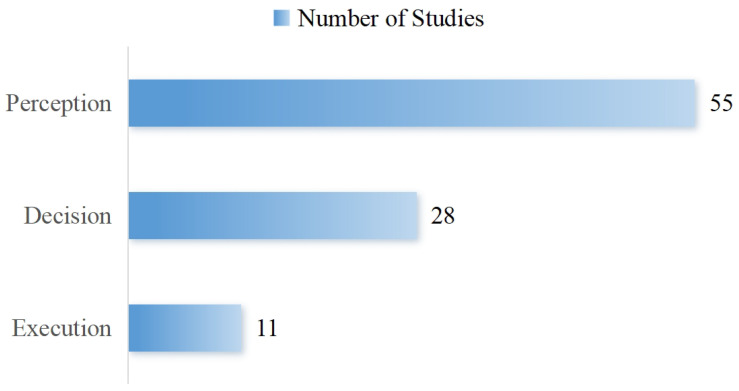
Distribution of included studies across three AI system components.

To make sure all relevant studies are included, we reviewed the reference lists of all included articles and the “Cited by” sections of key landmark papers, applying the same inclusion criteria to identify additional eligible studies. Figure 2 shows the entire literature screening process. In the end, we identified 89 publications that met all our inclusion criteria. These studies form the basis of our review. Figure 3 breaks down how these studies are distributed across the three core components of AI systems. It should be noted that while the total number of unique included studies is 89, five studies were classified into multiple functional categories because their proposed frameworks explicitly bridge multiple domains. Consequently, the sum of the category counts in Figure 3 amounts to 94.

To provide a structured overview of the current landscape and enable readers to identify overarching technical and clinical patterns rather than isolated examples, we have systematically summarized the key characteristics of all included studies in Table 1. This comprehensive comparison details the task, target anatomy, imaging modality, dataset size, validation type, evaluation metrics, and clinical maturity level for each study. By explicitly mapping these multidimensional attributes, the table serves as a foundational roadmap for the in-depth technical analyses and clinical implications discussed in the subsequent sections.

## 3. Key Models

This section takes a thorough look at the key artificial intelligence models that drive advancements in AI-assisted orthopedic surgery. We emphasize their foundational principles, architectural innovations, and clinical applicability and focus on why specific model families are particularly suited to overcome the unique anatomical and procedural challenges inherent in orthopedic surgery. By highlighting concrete comparative insights, this section bridges foundational AI architectures with their broad clinical applications, serving as a conceptual foundation for the detailed systems analyzed in subsequent sections.

### 3.1. Convolutional Neural Networks for Visual Representation Learning

Convolutional Neural Networks (CNNs) serve as the foundation for visual representation learning. As demonstrated by Krizhevsky et al. [100], they utilize local receptive fields, parameter sharing, and hierarchical feature extraction to achieve representation of high quality. In orthopedic surgery, CNNs are fundamental for extracting hierarchical features from diverse medical imaging modalities. For example, Zhang et al. [13] widely adopted 3D CNN variants to process volumetric data for joint lesion assessments, while Bier et al. [38] utilized lightweight CNN architectures to provide the computational efficiency required for real-time feature extraction during intraoperative navigation.

### 3.2. RNNs and LSTMs for Temporal Sequence Modeling

When it comes to temporal sequence modeling, Recurrent Neural Networks (RNNs) model sequential data through persistent hidden states, while Long Short-Term Memory (LSTM) networks address gradient issues through gated memory mechanisms. Given the highly sequential nature of orthopedic procedures, LSTMs and bidirectional LSTMs (BiLSTMs) are crucial for modeling temporal dynamics. For instance, NISHIO et al. [14], Kadkhodamohammadi et al. [15] utilized them to analyze surgical workflows through intraoperative video streams, and Sun et al. [16], Ji et al. [44] processed time-series sensor data, such as force or electrical impedance, to monitor critical surgical states and prevent intraoperative errors.

### 3.3. Transformer for Global Context Modeling

The Transformer introduced by Vaswani et al. [101] leverages self-attention mechanisms to capture long-range dependencies. In computer vision tasks, Dosovitskiy et al. [102] applied the same method to visual data through the vision transformer (ViT) by modeling images as sequences of patches. Compared to CNNs, Transformers excel at capturing global contextual patterns. This comparative advantage is highly beneficial in orthopedics for understanding the complex spatial relationships between multiple interconnected structures. As a result, Transformer-based models have driven advancements in tasks requiring broad spatial awareness, such as the simultaneous segmentation of multi-bone joint structures by Liu et al. [19] and the robust alignment of multi-modal surgical images by Ye et al. [37].

### 3.4. Siamese Networks for Similarity Learning

As detailed by Gao et al. [103], siamese networks use subnetworks with shared weights to learn discriminative embeddings that are useful for similarity matching. In surgical navigation, matching preoperative 3D scans with intraoperative 2D views is a persistent challenge. Siamese networks address this by evaluating the similarity between different image domains, serving as a core mechanism for cross-modal image registration in the approach by Zou et al. [28]. Additionally, Dunnhofer et al. [17] showed that their capacity for similarity learning enables consistent tracking of surgical targets or instruments even when densely annotated data is limited.

### 3.5. U-Net and Variants for Semantic Segmentation

The U-Net architecture proposed by Ronneberger et al. [104] established a standard encoder–-decoder framework for semantic segmentation using symmetric pathways. The U-Net family remains the gold standard for anatomical segmentation in orthopedics. Its defining feature—skip connections—is highly effective at preserving fine spatial details, making it uniquely suited for delineating critical soft tissues with ambiguous boundaries, such as cartilage and neural structures studied by Jonmohamadi et al. [21], Lee et al. [26]. Furthermore, Li et al. [27] demonstrated that advanced variants with attention mechanisms further improve performance by isolating surgical instruments from complex anatomical backgrounds.

### 3.6. YOLO Family for Object Detection

YOLO (You Only Look Once) performs end-to-end object detection by using direct regression over a dense grid. Unlike region-proposal-based models, YOLO’s single-stage detection architecture provides the ultra-low latency necessary for real-time intraoperative execution. This speed advantage makes the YOLO family the dominant choice for highly dynamic surgical tasks, including the real-time tracking of surgical instruments by Cho et al. [42] and the rapid localization of critical anatomical structures to prevent iatrogenic injuries during minimally invasive procedures by Lu et al. [20], Cui et al. [43].

### 3.7. Reinforcement Learning for Decision-Making

Reinforcement Learning (RL) focuses on optimizing sequential decision-making. It learns by interacting with the environment and receiving reward feedback. Deep RL (DRL), developed by Mnih et al. [105], extends this framework to address high-dimensional sensory inputs. In orthopedic decision systems, RL shifts the paradigm from static, rule-based planning to dynamic optimization. By treating surgical pathfinding as an environment-interaction problem, Zhang et al. [4], Cui [68] demonstrated that DRL agents can automatically optimize surgical trajectories within complex 3D anatomical spaces. Furthermore, Geng et al. [33] proved RL to be effective in automating complex spatial registration strategies.

## 4. Perception Systems in AI-Assisted Orthopedic Surgery

**Figure 4:**
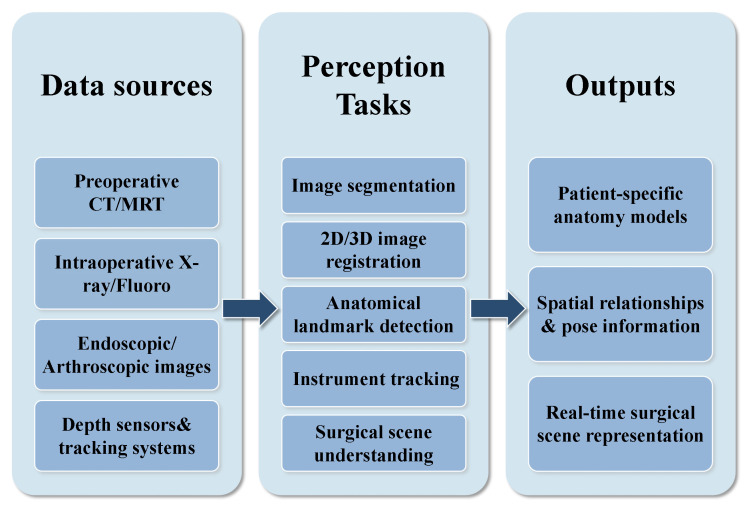
Data sources, core tasks and outputs of perception systems in AI-assisted orthopedic surgery.

Perception systems form the foundation of AI-assisted surgery. These systems extract and analyze anatomical information from various medical images and sensor data. Through this interpretation, models can understand the complex structures of the human body during orthopedic surgery. As shown in Figure 4, these systems provide a precise anatomical basis. The information then supports all subsequent surgical decisions and executions. This section examines nine core research areas within perception systems. These topics range from fundamental medical image segmentation and registration to advanced anomaly detection and surgical phase recognition. Perception systems use AI technologies like deep learning and computer vision to improve traditional orthopedic surgery. Specifically, they solve common problems in anatomical visualization, real-time navigation, and precise localization. This section begins with basic image segmentation techniques and then moves toward more complex multi-modal information fusion and real-time perception capabilities. This progression builds a hierarchical technical framework and provides the necessary groundwork for precise and intelligent AI-assisted orthopedic surgery.

**Figure 5:**
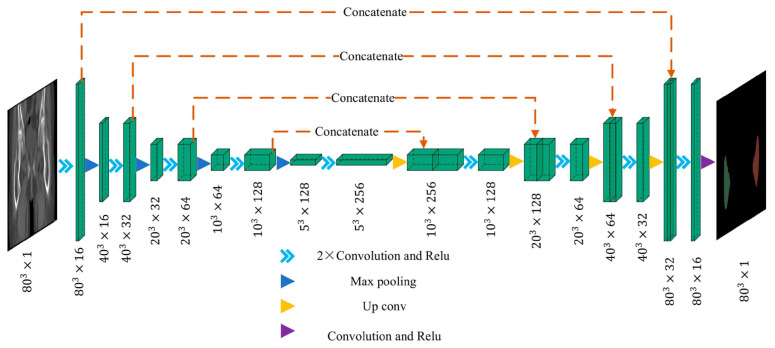
A representative methodology visualization of a perception system. Reproduced from Liu et al. [18] under the terms of the Creative Commons CC-BY 4.0 license.

To provide a concrete clinical example of these perception mechanisms in practice, Figure 5 illustrates a deep learning-based framework for segmentation tasks. This visualizes how raw CT data is automatically processed to isolate specific structures.

### 4.1. Medical Image Segmentation

As a fundamental anatomical prerequisite for AI-assisted orthopedic surgery, medical image segmentation enables the extraction of precise structural information from imaging data. This information then provides critical guidance for surgical practice, such as preoperative planning, implant placement, and real-time surgical navigation. Recent advances in deep learning have changed how models segment bone structures, including vertebrae and long bones. Meanwhile, soft tissue segmentation handles the specific challenges of low-contrast structures like ligaments and cartilage. Both types of segmentation are vital for intelligent orthopedic care.

#### 4.1.1. Bone Structure Segmentation

Bone structure segmentation is an essential foundational task in AI-assisted orthopedic surgery. It supports surgical planning, implant placement, and intraoperative navigation with clear anatomical information. Early research here focused on building baseline performance through deep learning models. Kuok et al. [3] proposed FC-DenseNet to segment vertebrae from 3D CT images. It demonstrated that dense connectivity boosts segmentation accuracy by keeping gradient information across layers. This work laid the groundwork for subsequent studies on more complex anatomical structures. Building on this foundation, Liu et al. [18] applied a 3D U-Net to femoral segmentation. This method was used to measure postoperative implant displacement, and the 3D architecture was better than 2D approaches at capturing volumetric bone structures. This capability was critical for accurately assessing the position of the implant relative to the femoral neck. However, both of these early studies relied on large, manually annotated datasets, which are expensive and time-consuming to create.

A significant advancement came with the DDA-Transformer introduced by Liu et al. [19]. This model introduced dual-domain attention mechanisms for the simultaneous segmentation of the femur, tibia, patella, and fibula in knee CT images. It addressed the challenge of segmenting multiple interconnected structures in a single pass. It is essential for the planning of robotic-assisted total knee arthroplasty (TKA). Notably, rather than relying solely on retrospective datasets, this method was validated through a multi-center prospective study. By achieving a mean Dice Similarity Coefficient (DSC) exceeding 0.98, the AI-generated models provided accuracy equivalent to manual segmentation while significantly reducing preoperative processing time, demonstrating a high degree of clinical readiness for real-world deployment.

For spinal applications, Lu et al. [20] developed a cascaded deep learning workflow. This system integrates localization, segmentation, and labeling for fully automated vertebra analysis. This three-stage pipeline uses YOLOv7 for vertebra localization. It transforms 3D localization into 2D localization via DBSCAN clustering to improve speed and accuracy. Then, a 3D U-Net with attention mechanisms enhances the segmentation of complex vertebral boundaries. In the end, a hybrid architecture of ResNet and the Transformer fuses local and global features for robust vertebral labeling.

#### 4.1.2. Soft Tissue Segmentation

Soft tissue segmentation presents greater challenges than bone segmentation. They stem from the inherent variability and low contrast of soft tissues in medical images. Early research in this field focused on knee arthroscopy applications. Jonmohamadi et al. [21] developed the first automatic segmentation method for several key knee structures. It used U-Net and U-Net++ architectures to segment the femur, Anterior Cruciate Ligament (ACL), tibia, and meniscus. Imbalanced arthroscopic data posed a key challenge for the study, with the femur appearing significantly more often compared to other anatomical structures. To tackle this problem, these models are adapted by combining the Dice coefficient loss and cross-entropy loss. Due to the nested dense skip connections of U-Net++, it showed better segmentation performance for specific structures. But on the other side, these connections also increased the total training time.

Building on knee arthroscopy segmentation, Ali et al. [22] used a standard UNet architecture for multi-spectral scene segmentation. This study demonstrated that models could effectively distinguish between different soft tissue types in endoscopic images. Ali and Pandey [23] improved this work through the development of ArthroNet. This model integrates depth estimation with semantic segmentation. Through this combination, the system is able to generate 3D segmented maps of knee structures. It overcomes the limitations of traditional endoscopic views that lack spatial depth cues.

The introduction of 4D ultrasound imaging created new opportunities for dynamic soft tissue analysis in orthopedic surgery. Many studies focused on femoral cartilage segmentation using these 4D images. This specific task is critical for guiding robotic knee arthroscopy. Precise segmentation allows for better visualization of moving joint structures during a procedure. For instance, Antico et al. [24] developed a U-Net-based model designed for this task. And high Dice coefficients are achieved by applying this model to in vivo data. Antico et al. [25] refined this approach by implementing a Bayesian CNN that utilizes Monte Carlo dropout. This model quantifies segmentation uncertainty, providing a measure of reliability for the results. Such transparency is vital for clinical decision-making, as the confidence level in these segmentations directly affects surgical outcomes.

The neural tissue segmentation in biportal endoscopic spine surgery developed by Lee et al. [26] represents a notable departure from joint surgery applications. In this study, a U-Net-based model was developed for segmenting neural tissues in endoscopic images. The core goal here was to address the unique challenges inherent to spinal endoscopy, where the visual field is limited, and tissue boundaries tend to be indistinct. The model demonstrated high accuracy in identifying neural structures. These findings confirm the robust generalizability of the U-Net for segmentation tasks.

### 4.2. Image Registration

2D/3D image registration is a vital module of orthopedic surgical navigation. This process enables the alignment of preoperative 3D images with intraoperative 2D data. More specifically, image registration allows surgeons to match 3D scans against 2D fluoroscopic or ultrasound images. It ensures that preoperative plans correspond accurately to the patient’s position during surgery.

For rigid medical image registration, an innovative approach developed by Zou et al. [28] integrates fully convolutional networks (FCN) for interest point detection and CNN for feature processing. Two methods are proposed in this paper. The proposed FIP-CNNF method employs FCN for pixel-level interest point detection. It utilizes an improved Siamese network for feature detection, description, and matching. Furthermore, its variant called TrFIP-CNNF incorporates transfer learning and fine-tuning strategies to tackle the issue of data scarcity. This learning-based framework demonstrates improved performance for multi-modal image registration. Yu et al. [29] took a different approach to vertebra matching. This method uses a detection-based framework that relies on Faster-RCNN. At the same time, it is also combined with a modified generalized Hough transform. These two components work together to ensure the images align correctly. This method leveraged object detection to reduce the search space required for registration. It improves robustness against image artifacts and performs effectively when dealing with partial occlusions.

Fan et al. [30] built upon these early learning-based approaches to address challenges in C-arm cone-beam computed tomography (CBCT). The study specifically focuses on the problem of severely truncated data. To solve this, it developed two AI-based methods that facilitate the detection and recovery of fiducial markers. The direct detection method uses a U-Net for segmentation and a ResNet for depth detection. And the recovery method employs FBPConvNet and Pix2pixGAN to restore marker shape and intensity. The recovery method shows superior robustness, while the direct method offers faster computation. One key innovation lies in the task-specific data preparation strategy. It directs model learning toward markers to enhance robustness against truncation.

For ultrasound-based navigation in scaphoid fracture surgery, Broessner et al. [31] developed a fully automatic, nearly real-time system to eliminate fluoroscopy and manual interaction. This two-stage approach combines DeepLabv3+ and PRNet to achieve image registration. First, DeepLabv3+ is used to segment the carpal bones in ultrasound images. Then, PRNet performs partial-to-full point set registration. This step aligns point sets derived from intraoperative ultrasound with preoperative CT models. PRNet addresses the limited field-of-view constraint in ultrasound images by non-bijective correspondences. It enables the system to attain precise registration without manual seed point placement.

In knee arthroscopy navigation, Banach et al. [32] introduced a single-image PoseNet-based framework for 6-DoF arthroscopic camera localization relative to bone markers. This single-image localization approach eliminates the need for temporal tracking. It addresses the limitations of traditional SLAM methods, which often fail in water-filled and specular arthroscopic environments. The model achieves real-time implementation. And it also maintains high performance across various knee flexion angles.

For CT to X-ray registration, Geng et al. [33] introduced CT2X-IRA. It uses a domain-cross deep reinforcement learning agent with multi-scale-stride approach. By learning registration strategies directly from data, it eliminates the need for manual feature engineering and optimization hyperparameter tuning. As a result, CT2X-IRA demonstrated superior performance in both accuracy and speed. It proved particularly effective when handling challenging cases involving significant image noise. In reverse, for X-ray to CT registration, Shrestha et al. [34] proposed a U-Net-based scene coordinate regression method for fully automatic 2D-3D registration. By regressing dense scene coordinates alongside uncertainty estimation, this landmark-free approach removes the necessity for manual annotation. And it was trained on simulated X-rays to establish these spatial relationships. The integration of uncertainty filtering ensures robust registration even when encountering extreme or partial views.

In the field of spine surgical navigation, Chen and Zhang [35] introduced a coarse-to-fine optimization framework using the Covariance Matrix Adaptation Evolution Strategy (CMA-ES). The study demonstrates that optimization-based methods can reach sub-millimeter accuracy. However, achieving this high level of precision requires the allocation of sufficient computational resources. In minimally invasive pelvic surgery, Ju et al. [36] designed a multi-module AI framework for 2D/3D registration. It integrates deep learning for feature extraction with an optimization-based refinement stage. In this way, the framework captures the rapid processing power of neural networks alongside the fine-tuned precision of traditional optimization. This hybrid approach successfully addressed the trade-off between speed and accuracy.

XPE-ST, introduced by Ye et al. [37], represents another significant development for real-time image registration. It was built with an enhanced Swin Transformer framework. To enhance sensitivity to subtle pose changes, XPE-ST utilizes a dual-channel input design. This architecture enables the direct learning of feature and pose differences. Moreover, it integrates squeeze-and-excitation attention mechanisms with feature pyramid networks to improve feature consistency and noise robustness. The large-scale datasets used are facilitated for creation by the implementation of GPU-accelerated digitally reconstructed radiograph generation.

### 4.3. Anatomical Landmark Detection and Localization

Precise surgical planning relies heavily on anatomical landmark detection and localization, which are also essential for implant placement and intraoperative guidance. For pelvic applications, Bier et al. [38] introduced a sequential CNN framework to detect 23 clinically significant anatomical landmarks. This system identifies these points in X-rays captured from arbitrary viewing angles by utilizing a multi-stage convolutional and pooling process to generate belief maps for each landmark. The model combines local image features with predictions from previous stages; as a result, the sequential refinement effectively resolves spatial ambiguities. Moreover, data scarcity is addressed by generating synthetic data, allowing the model to generalize directly to real clinical X-rays without additional training. The outputs of this model support registration initialization without the need for manual calibration.

Wang et al. [39] addressed another specific clinical need by developing a deep learning method to locate intramedullary nail holes on 2D calibrated fluoroscopic images, which are standard in orthopedic interventions. The study demonstrated that AI models can accurately identify small, low-contrast features within these scans. Lee et al. [40] presented a unique application of landmark detection, utilizing MediaPipe Pose for clinical evaluation. Their method extracted 3D body landmarks and gait parameters to assess patients after total hip arthroplasty, enabling a detailed analysis of physical recovery through automated motion tracking. The use of consumer-grade cameras allows this method to be adopted for widespread clinical application. Additionally, YOLOv5-6D, proposed by Viviers et al. [41], advanced the field by applying 6-DoF instrument pose estimation to variable X-ray imaging geometries. It integrates object detection with pose regression to enhance tracking capabilities, allowing the simultaneous estimation of both the location and the orientation of surgical instruments.

### 4.4. Surgical Instrument Recognition and Tracking

Surgical instrument recognition and tracking are critical for robotic-assisted surgery, enabling real-time monitoring of instrument position and state. Early work in this field focused on single-modality approaches. An automated method was developed by Cho et al. [42] to detect surgical instrument tips in biportal endoscopic spine surgery. This model combines RetinaNet and YOLOv2, optimizing the margin size of bounding boxes surrounding the annotated tip coordinates to improve localization accuracy. This marked the first application of deep learning for instrument tip detection in this specific surgical context.

As robotic surgery became more complex, the need for multi-modal sensing emerged. Ji et al. [44] introduced an LSTM-FCN model designed to predict lamina breakthrough during spinal surgery by utilizing electrical impedance monitoring to track changes in tissue properties. By combining temporal sequence modeling with spatial feature extraction, the model effectively detects the transition from bone to soft tissue, demonstrating that electrical impedance provides valuable complementary information to visual or acoustic sensing. In the realm of real-time articulated joint detection, Sun et al. [45] developed a lightweight bi-branch deep neural network for minimally invasive surgical robots. Based on the BiSeNet V2 backbone, the model integrates two distinct task branches—one for articulated joint detection and the other for instrument classification. This dual-branch design facilitates efficient feature sharing, balancing real-time performance with high detection accuracy.

For ultrasound-based tracking, Lan et al. [46] presented a cascaded U-Net framework for real-time bone tracking, utilizing A-mode ultrasound signals to monitor bone surfaces during surgery. This method demonstrated that ultrasound provides highly accurate tracking without the risks associated with ionizing radiation. Meanwhile, an attention-based CNN-LSTM model developed by Sun et al. [47] used acoustic features for bone-milling state recognition. In this manner, surgeons can ascertain the relative positional relationship between bones and surgical instruments. Sun et al. [16] further integrated vibration and sound signals with an LSTM for milling state identification in robot-assisted cervical laminectomy, showing that multi-modal sensing provides more robust state recognition than single-modal approaches.

Recent work has investigated surgical instrument tracking as a segmentation task, focusing on improving accuracy for specific applications. Several studies by Dunnhofer et al. [17], Li et al. [27], Zhang et al. [48], Nwoye and Padoy [49], Xu et al. [50] have developed UNet++ models with attention gates for spinal surgical instrument segmentation. These studies show that attention mechanisms boost segmentation accuracy in complex scenarios where instruments and tissue overlap. A novel approach, MSDE-Net introduced by Yang et al. [51], uses a multi-scale dual-encoding network to capture both local and global features. Finally, Sheng et al. [52] framed the problem as a graph partitioning task. This label-free method leverages ViT-based self-supervised pre-trained models (specifically DINO) to extract high-level features. After feature extraction, the system constructs a graph where individual pixels serve as nodes, with edge weights defined by the cosine similarity between corresponding features. This approach handles all four categories of surgical instrument segmentation tasks without requiring human annotations.

### 4.5. 3D Reconstruction and Visualization

3D reconstruction and visualization play a crucial role in modern orthopedic surgery by providing surgeons with detailed anatomical models essential for preoperative planning and intraoperative guidance. Jonmohamadi et al. [53] developed an AI system for 3D semantic mapping using arthroscopy images. The framework combines out-of-distribution pose and depth estimation with in-distribution segmentation training, addressing the inherent challenges of reconstructing 3D models from endoscopic data where 2D images provide only a limited field of view.

Another clinical application of 3D reconstruction was presented by Abel et al. [54], who validated deep-learning-reconstructed lumbar spine 3D MRI as a radiation-free alternative to CT for spine surgery planning. The study proved that the reconstructed MRI provides geometric measurements comparable to those obtained via CT. This high level of correspondence is particularly vital for planning pedicle screw placement. By utilizing this method, clinicians can maintain surgical accuracy while reducing patient exposure to radiation.

### 4.6. Anomaly Detection and Disease Diagnosis

Anomaly detection and disease diagnosis represent critical applications of AI within orthopedic surgery, enabling the early detection of musculoskeletal conditions essential for improving patient outcomes.

A notable application of AI in clinical workflow automation was presented by LewandrowskI et al. [55], who evaluated deep learning algorithms for automated spine MRI reporting. Their study demonstrated that these algorithms can accurately detect and classify common spinal pathologies, significantly reducing radiologist workload and enhancing reporting consistency. Moreover, Cui et al. [43] addressed a critical need in spinal surgery by developing a YOLOv3-based computer-aided detection system for the real-time recognition of the nerve and dura mater during percutaneous transforaminal endoscopic discectomy (PTED). The study shows that AI-driven visualization helps surgeons avoid iatrogenic nerve injury.

As deep learning models became more complex, researchers began exploring multi-modal and multi-level classification approaches. Zhang et al. [13] introduced R-AlexNet, a model specifically tailored for the multi-level classification of knee cartilage lesions using multimodal MRI, demonstrating that the integration of multiple MRI sequences enhances classification accuracy. Similarly, a dual-stream parallel model named LC-DSPNet was developed by Fang and Wang [56] for cartilage injury diagnosis based on local centroid optimization, achieving high accuracy in grading cartilage injuries from MRI images.

Expanding spinal pathology detection to X-ray imaging, Ghauri et al. [57] developed a cloud-based deep learning approach. This framework combines the strengths of EfficientNet and ResNet to detect and classify three common degenerative spinal conditions: osteophytes, spinal implants, and foraminal stenosis. It adopts a cost-effective cloud-based implementation scheme and features strong robustness to heterogeneous X-ray data.

For spinal applications, an enhanced ViT was introduced by Xiang et al. [58] to grade nucleus pulposus degeneration using ultrasound signals. To optimize the model for ultrasonic data, the researchers introduced a specialized patch tokenizer and utilized channel-independent training. These innovations enable the model to better capture the subtle acoustic patterns and textural variations indicative of intervertebral disc decay. For knee ligament injury diagnosis, a refined detection and classification method was developed by Voinea et al. [59]. The study adapted ResNet-101 to process 3D volumes constructed from stacks of 2D MRI slices, enabling the accurate classification of three severity levels of ACL injury. A novel two-stage training process combined with tailored data augmentation was proposed to handle the spatial complexity of knee ligaments. Finally, Burlison et al. [60] validated an AI-based Active Appearance Model (AAM) algorithm to generate CT-derived B-scores, a quantitative imaging biomarker for measuring osteoarthritis (OA) severity. The AI framework provides objective, automated measures of OA that correlate strongly with traditional MRI-based assessments and clinical risk factors.

### 4.7. Image Quality Assessment

Image quality assessment plays a vital role in maintaining the reliability of AI-driven orthopedic surgery systems. When image quality is poor, the system may produce inaccurate results during segmentation, registration, or detection tasks. These errors can compromise the overall surgical outcome. To address this matter, a one-class Gaussian Process Deep Neural Network (GPDNN) with DenseNet backbone was presented by Antico et al. [61]. The model enables automatic ultrasound image quality assessment by focusing on femoral cartilage boundary detection. The study confirmed that AI models can evaluate image quality in real time.

Wang et al. [62] adopted a different strategy by developing a hybrid super-resolution model based on stereo attention. This model enhances the resolution of surgical images to facilitate more precise anatomical structure detection and segmentation. By utilizing a stereo attention mechanism, the model effectively captures spatial dependencies between stereo image pairs. It yields superior super-resolution performance when compared to traditional single-image methods.

### 4.8. Tissue Identification and Classification

Tissue identification and classification are essential for preventing iatrogenic injury during orthopedic surgery. This task becomes particularly critical in minimally invasive procedures, where surgeons experience limited direct tactile feedback. Gunaratne et al. [63] combined diffuse reflectance spectroscopy (DRS) and autofluorescence spectroscopy with machine learning to identify human joint tissue. This approach proved that spectroscopic data can accurately distinguish between different tissue types during surgical procedures. It provided a reliable framework for real-time tissue classification.

Researchers have continued to build on this foundation. Specifically, Gunaratne et al. [64] explored how machine learning can classify joint tissue using DRS data. This study combined DRS with Multiclass Fisher’s Linear Discriminant Analysis to reduce dimensionality. Then, it used Linear Discriminant Analysis for the final classification. These methods allowed the model to process complex data more efficiently. The study defined optimal spectral parameters to help with clinical translation. And it also showed that the model maintains robust performance even when class imbalance issues occur. As for spinal endoscopic surgery, a YOLOv3 model was developed by Cui et al. [65] to recognize the nerve and dura mater. This system addresses the problems caused by narrow working channels and limited visibility. These challenges force surgeons to rely heavily on their own experience. This study treats detection as a regression task to ensure fast inference. It pioneered the use of deep learning for tissue recognition in spinal endoscopy. Most recently, this field has been advanced by a forward-oriented endoscopic ultrasound system presented by Yao et al. [66]. This system uses DenseNet and ViT for spinal tissue classification. The combination of endoscopic ultrasound and AI models provides real-time tissue identification during spinal surgery. It also improves both surgical safety and accuracy.

### 4.9. Surgical Phase Recognition

Surgical phase recognition is a critical component of context-aware surgical assistance systems, allowing AI algorithms to adapt to the intraoperative workflow in real time. NISHIO et al. [14] advanced this field with a Conv-LSTM network, which utilized multiple CNN backbones to recognize phases in unicompartmental knee arthroplasty (UKA). By relying on input from a wearable camera, the system ensures that the AI remains synchronized with the actual progress of the surgery, facilitating seamless assistance.

Kadkhodamohammadi et al. [15] expanded these applications to open orthopedic surgery by developing a privacy-compliant pipeline utilizing a head-mounted camera. This system integrates image capture, face anonymization, and surgical phase recognition, employing a CNN-LSTM model with SENet154 and a bidirectional LSTM to recognize phases in open total knee arthroplasty (TKA). By adopting a surgeon-centric capture approach, the framework avoids disrupting the OR team. Consequently, this method enables automated workflow analysis and enhances surgical training for open procedures.

### 4.10. Summary: The Evolving Landscape of Surgical Perception

The landscape of surgical perception has undergone a significant paradigm shift. Technically, the field has transitioned from basic CNNs to more sophisticated transformers that exhibit a superior understanding of spatial and temporal contexts. For instance, Liu et al. [19] utilized a DDA-Transformer to demonstrate the efficacy of this architecture for complex tasks such as knee segmentation. Furthermore, there is a clear move toward multi-modal sensing rather than relying solely on computer vision. Recognizing that camera views are frequently obstructed, Sun et al. [16] integrated vibration and acoustic signals to maintain surgical tracking even in low-visibility conditions. Accordingly, the evaluation of these systems has evolved from assessing accuracy on static datasets to validating performance within the dynamic environment of the operating room.

Despite this progress, a major challenge remains: model generalization. Current models often fail when encountering rare pathologies or unfamiliar instruments. This limitation stems from a scarcity of diverse, labeled clinical datasets. Robust perception is a prerequisite for reliable decision-making; as Hopkins et al. [67] demonstrated with their infection prediction model, substandard input data inevitably leads to erroneous recommendations. This highlights that perception and decision-making are inextricably linked; if one component fails, the entire system is compromised. Moving forward, the research focus should shift from marginal improvements in segmentation accuracy toward enhancing system reliability in unexpected clinical scenarios. Addressing the data shortage through synthetic data generation or cross-institutional collaboration, alongside developing evaluation metrics for clinical utility rather than just technical performance, will be essential for the next generation of surgical AI.

## 5. Decision Systems in AI-Assisted Orthopedic Surgery

**Figure 6:**
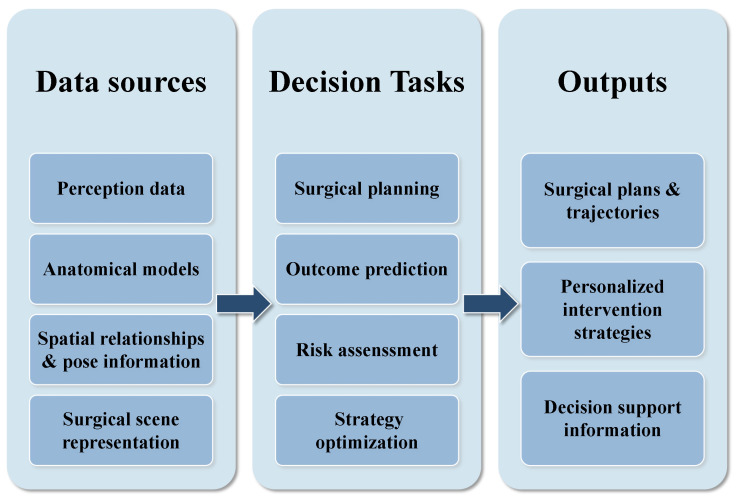
Data sources, core tasks and outputs of decision systems in AI-assisted orthopedic surgery.

Decision systems represent the cognitive core of AI-assisted surgery, translating raw anatomical data and intraoperative perceptions into actionable surgical plans. As illustrated in Figure 6, these systems serve as the critical bridge between data acquisition and clinical execution. This section examines seven key areas where decision-making frameworks are transforming orthopedic practice, ranging from preoperative planning and implant selection to risk assessment and patient communication.

By leveraging machine learning, these tools address a fundamental limitation of traditional surgery: the heavy reliance on an individual surgeon’s experience or subjective intuition. Rather than adopting a generalized approach, these systems facilitate the creation of highly personalized surgical strategies. The ultimate objective is to establish a comprehensive framework that supports clinicians through every phase of the perioperative workflow, thereby enhancing the precision, safety, and reproducibility of orthopedic interventions.

**Figure 7:**
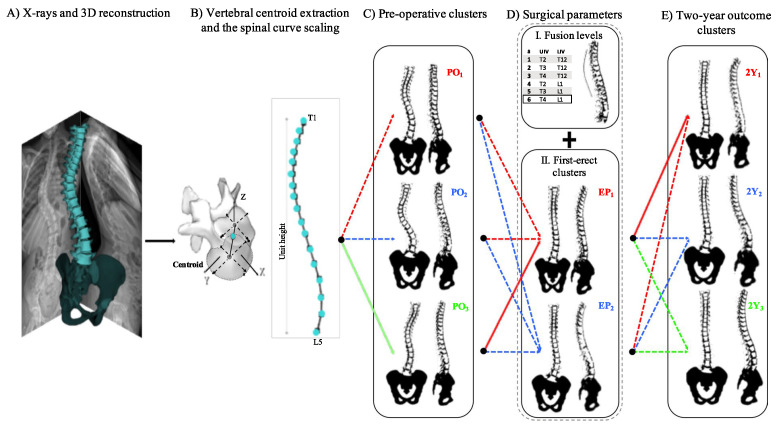
A representative methodology visualization of a decision system. Reproduced from Pasha and Flynn [76] under the terms of the Creative Commons CC-BY 4.0 license.

As a tangible example of clinical decision support, Figure 7 depicts a data-driven framework utilized for classifying 3D spinal curves. This illustrates the translation of extracted anatomical parameters into actionable prognostic insights and surgical outcome predictions.

### 5.1. Surgical Planning and Optimization

Effective preoperative planning is arguably the most critical stage of orthopedic surgery, as it dictates patient safety and determines long-term recovery outcomes. AI-driven path planning technologies have significantly advanced the field by calculating precise trajectories that avoid sensitive structures, such as nerves and blood vessels. This technology has evolved beyond static preoperative preparation; it can now adapt dynamically during the surgical procedure. Simultaneously, intelligent systems for implant selection have improved substantially. By analyzing patient-specific anatomy, these tools facilitate the selection of implants that offer superior fit and increased longevity. Together, these innovations have transformed surgical preparation into a process driven by precision and empirical data.

#### 5.1.1. Path Planning

Path planning is a fundamental component of surgical decision systems, enabling surgeons to identify optimal trajectories while avoiding critical anatomical structures. Early research in this field utilized reinforcement learning (RL) for preoperative path planning to automate the selection of safe entry points and trajectories. For instance, a Q-learning approach was introduced by Zhang et al. [4] for 3D preoperative path planning in anterior spinal surgery. This study demonstrated that RL can generate safe, optimal paths using CT images by training the model to navigate around sensitive anatomy. This work established a foundation for subsequent developments in the field.

As techniques evolved, researchers adopted Deep Q-Networks (DQNs). Cui [68] showed that these models manage complex policies more effectively than standard Q-learning, enabling real-time optimization during the actual procedure. More recently, the research priority has shifted toward radiation-free methods. A significant advancement is the SafeRPlan framework introduced by Ao et al. [69], which utilizes ultrasound for pedicle screw placement. This framework explicitly incorporates safety constraints into the algorithm to ensure patient well-being while eliminating radiation exposure.

#### 5.1.2. Implant Selection and Placement

Selecting the appropriate implant and positioning it correctly is critical in orthopedic surgery, as it directly determines implant longevity and patient recovery. Early efforts focused on automating the preoperative workflow. Fauser et al. [70] successfully combined deep learning with trajectory planning to map out access to the cochlea and internal auditory canal. By utilizing a hybrid approach combining 2D U-Nets with shape models, this system handled the complex segmentation of small structures more efficiently and accurately than manual methods.

For spinal applications, Siemionow et al. [71] validated a neural network for autonomous lumbar pedicle screw planning. This system integrates semantic segmentation with anatomical landmark localization. The end-to-end model calculates the optimal screw length, diameter, and angulation without human intervention. By building on previous solutions for autonomous spine segmentation, it provides clinically appropriate screw parameters that align precisely with the lumbar pedicle anatomy.

Moreover, researchers such as Wang et al. [72] have begun pairing AI models with 3D-printed guides to manage complex cases of bone cancer. In a prospective clinical trial, this integrated system analyzed the patients’ unique anatomy to identify optimal entry points for screws, achieving a 100% success rate in screw placement with a mean deviation of less than 2 mm. These results provide strong clinical evidence for the effectiveness of AI-driven planning in complex oncological reconstructions, separating its technical promise from validated real-world execution.

AI models have also found a significant role in robotic knee replacements. Novel architectures like the DDA-Transformer introduced by Liu et al. [19] can map the femur, tibia, and patella simultaneously. This provides the precise anatomical data required for correct implant alignment, a decisive factor in the long-term success of knee arthroplasty.

### 5.2. Surgical Risk Prediction and Assessment

Predicting risks beforehand allows surgeons to make more informed clinical decisions. One specific area of interest is determining the optimal level of invasiveness for spine surgery. Campagner et al. [73] first utilized machine learning to estimate the invasiveness of lumbar fusion procedures, helping clinicians tailor their approach to the specific patient and avoid unnecessary trauma. However, risks are not limited to the patient; the physical toll on the surgeons themselves is often overlooked. To address this, Sánchez-Guillén et al. [74] developed a calculator that predicts musculoskeletal injuries in medical staff. By identifying these occupational hazards early, hospitals can implement more effective prevention strategies.

Moreover, AI is increasingly utilized to communicate these risks to patients. A recent study by Dasci et al. [75] compared ChatGPT and Google in their ability to answer patient questions regarding robotic hip replacements. The analysis found that the AI provided accurate and accessible information, suggesting that chatbots could play a major role in helping patients understand the benefits and risks of their procedures.

### 5.3. Prognostic Outcome Prediction

Prognostic outcome prediction is critical for guiding treatment decisions and managing patient expectations in orthopedic surgery. In cases of adolescent scoliosis, for instance, a data-driven framework developed by Pasha and Flynn [76] has demonstrated superior accuracy in classifying spinal curves compared to traditional manual methods. For high-risk conditions such as spinal metastasis, a precise assessment of mortality risk fundamentally alters care planning. Algorithms validated by Karhade et al. [77] on over 4000 patients can now predict six-week mortality with reliable accuracy, assisting clinicians in making complex palliative care decisions. Similarly, Toyoda et al. [78] developed a CHAID decision tree model to identify patients predisposed to unfavorable outcomes following lumbar decompression. This allows surgeons to identify candidates who may not derive significant benefit from operative intervention.

For lumbar disc procedures, Chen et al. [79] utilized machine learning to identify recurrence risks by analyzing specific factors such as BMI and disc height. Furthermore, deep learning models—specifically those capable of processing sequential data—have outperformed standard machine learning methods in predicting gait patterns following knee replacement. Zhang et al. [80] highlighted that these models are particularly effective at capturing the complex, changing dynamics of gait associated with implant rotation, providing higher-quality data for surgical planning.

### 5.4. Surgical Decision Support

Surgical decision support systems provide clinicians with evidence-based recommendations to improve decision-making in orthopedic surgery. Starting with foundational classification models, Ahammad et al. [81] developed a filter-based multi-level segmentation model to classify spinal cord disorders. This system leverages an improved Random Forest (IRF) classifier. It incorporates several novel preprocessing methods to handle missing values and uses multi-level Expectation-Maximization (EM) clustering for feature selection. The integration of AdaBoost with IRF further enhances the robustness of the classification.

Liao et al. [82] built on this foundation by comparing multiple machine learning models to predict the optimal selection of vertebroplasty methods. This approach highlights the potential of AI to reduce treatment variability and improve patient outcomes. Similarly, a Message Passing Neural Network (MPNN) model was developed by Cavazos et al. [83] to predict the risk of blood transfusion in primary total knee arthroplasty. By identifying high-risk patients, the system enables targeted blood management strategies.

AI-generated 3D fusion images have also improved the determination of surgical indications. Specifically, Yamada et al. [84] used these images to assess full endoscopic discectomy at lumbosacral disc levels. This approach increases the accuracy of surgical decisions and helps reduce unnecessary surgeries. As a safety improvement, Abel et al. [54] replaced CT scans with deep-learning reconstructed MRIs, which provide equivalent planning accuracy without radiation exposure. This method serves as a viable alternative to CT scans for spine surgery planning.

Furthermore, Liu et al. [85] introduced a patient-specific musculoskeletal modeling framework to enhance personalized planning. This system is designed for pelvic fracture reduction planning, enabling personalized preoperative plans based on the unique anatomy of each patient. Other researchers have focused on complementing preoperative planning with intraoperative guidance. Thibeault et al. [86] developed an Articulated Neural Kernel Fields framework to forecast the shape of the standing spine during the operation. It allows surgeons to optimize surgical posture based on the predicted final standing shape.

In the domain of post-surgical assessment, Lee et al. [40] validated AI pose estimation as an objective evaluation tool. The study focused on functional recovery after total hip arthroplasty. The researchers used MediaPipe Pose to extract gait parameters, which were then correlated with Harris hip scores. This method serves as a valuable addition to traditional clinical assessments. Researchers have also extended this approach to rehabilitation; for instance, Kim et al. [87] developed an XGBoost model utilizing IMU sensors to classify elbow rehabilitation postures. Moreover, Nonnenmacher et al. [88] developed a tree model to predict when patients can return to sports after a periacetabular osteotomy. By using these predictive models, medical teams can provide more personalized and accurate recovery timelines.

### 5.5. Clinical Risk Stratification

Identifying high-risk patients early is essential for optimizing the allocation of hospital resources. Hopkins et al. [67] developed a deep learning model specifically to predict surgical site infections following posterior spinal fusion. By analyzing dozens of clinical variables, the system successfully identified patients most susceptible to complications, highlighting conditions such as HIV, multilevel fusions, and chronic lung failure as significant risk factors.

### 5.6. Patient Education and Communication

Patient education and communication are critical for improving outcomes in orthopedic surgery. These processes help patients understand their conditions and participate in shared decision-making. Subramanian et al. [89] evaluated how well ChatGPT could answer common questions regarding minimally invasive spine surgery. The study demonstrated that AI provides accurate and accessible information to patients, highlighting its potential to enhance patient education.

### 5.7. Telehealth and Remote Monitoring

Conducting orthopedic examinations via video consultation is inherently challenging because clinicians cannot physically palpate the patient. To address this limitation, Elmakias and Dabran [90] developed a system that utilizes VR sensors to precisely track a patient’s movements at home. Integrating this motion data into a machine learning model proved highly effective; the system detected neck pain and wrist injuries with high accuracy, demonstrating that remote examinations can achieve significant precision.

### 5.8. Summary: From Data to Actionable Intelligence

AI in orthopedics is a multifaceted framework, encompassing everything from surgical path planning and implant selection to preoperative risk prediction. The field has undergone a significant shift in methodology. Early efforts relied on rigid rules and optimization, such as the approach by Zhang et al. [4] using Q-learning to map spinal trajectories. Currently, the field has transitioned toward data-driven approaches. Hopkins et al. [67] demonstrated that deep neural networks now predict infection risks by analyzing clinical patterns that are too complex for traditional rule-based systems to identify.

However, a significant challenge remains: the black box problem. Many models provide recommendations without explaining the underlying reasoning, which can hinder a surgeon’s trust. Transparency is essential; as highlighted by Dasci et al. [75], even when a chatbot provides information to a patient, trust depends on understanding the source and logic of the answer. Another critical issue is that these models often lack uncertainty estimation, providing definitive answers without indicating the level of confidence.

In clinical settings where every decision involves a trade-off between risk and benefit, clinicians must know if the AI is certain or merely estimating. Furthermore, the quality of a decision is fundamentally limited by the input data; if a system fails to accurately identify anatomical structures, precise implant placement becomes impossible. Consequently, developing robust systems capable of processing noisy or incomplete data is essential. The future likely resides in the integration of hybrid models, which combine machine learning techniques with established medical expertise to produce tools that are both intelligent and practical for clinical application.

## 6. Execution Systems in AI-Assisted Orthopedic Surgery

**Figure 8:**
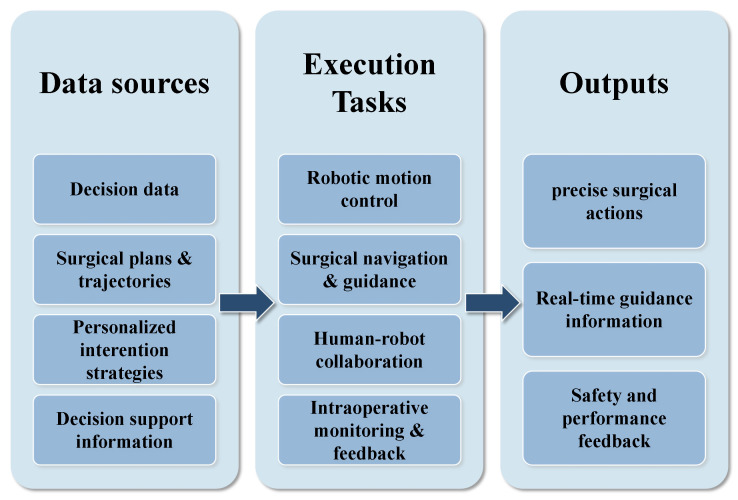
Data sources, core tasks and outputs of execution systems in AI-assisted orthopedic surgery.

Execution systems serve as the physical implementation layer of AI-assisted orthopedic surgery. They take the raw anatomical data from perception systems and the calculated plans from decision systems, translating them into precise physical operations. As shown in Figure 8, these systems act as the bridge between data analysis and surgical action. This section covers six core research areas, ranging from surgical robot control and augmented reality navigation to human–robot collaboration and emerging technologies. By leveraging robotics and computer vision, these execution systems address the fundamental limitations of traditional surgery—specifically the need for higher precision, real-time feedback, and minimally invasive capabilities. The discussion moves from the technical basics of robot control toward increasingly complex collaborative applications, outlining a framework that spans from technical implementation to clinical reality.

**Figure 9:**
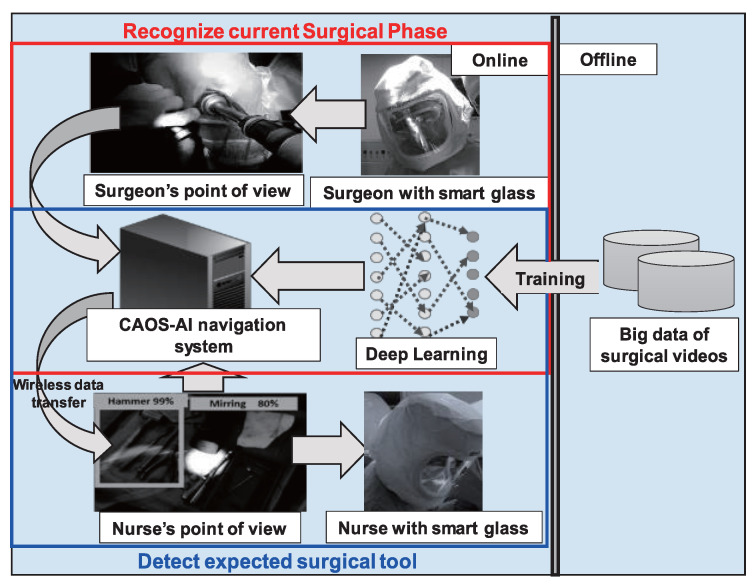
A representative methodology visualization of an execution system. Reproduced from NISHIO et al. [14] under the terms of the Creative Commons CC-BY 4.0 license.

To contextualize how these execution systems operate in a real-world surgical environment, Figure 9 demonstrates a surgical phase recognition system using a wearable camera. This highlights the real-time, context-aware integration of AI models directly into the surgical workflow.

### 6.1. Surgical Robot Control

Surgical robot control constitutes the core mechanism of execution systems, enabling precise and repeatable interventions that minimize collateral tissue damage. In minimally invasive knee surgery, Shen [91] integrated multiple control modes to allow the system to adapt to varying surgical tasks, significantly enhancing both operational flexibility and accuracy. A primary challenge in robotic orthopedics is the precise detection of bone breakthrough to prevent accidental over-penetration. Torun and Öztürk [92] demonstrated that machine learning methods are now capable of analyzing force and torque profiles to detect the moment the drill pierces the bone. This capability is particularly critical in spinal surgery, where direct visualization is often restricted.

For robotic laminectomy, Ji et al. [44] utilized an LSTM-FCN model for electrical impedance monitoring. By integrating temporal sequence modeling with spatial feature extraction, this system effectively predicts lamina breakthrough, thereby mitigating the risk of neural injury. Furthermore, contemporary robotic systems are being developed to emulate the tactile expertise of skilled surgeons. Li et al. [93] compared LSTM-based imitation learning with conventional impedance models to facilitate the transfer of force perception skills. By employing a dataset of expert grinding demonstrations synchronized with force, position, and velocity signals, the LSTM approach successfully captured critical temporal dependencies. This enables the robot to perform real-time, adaptive grinding that mimics human techniques, outperforming traditional fixed-mode operations.

### 6.2. Augmented Reality Navigation and Guidance

Augmented Reality (AR) provides a digital overlay that projects a patient’s internal anatomy directly onto the surgical field. While earlier methods relied on manual landmark placement, newer multi-modal, fiducial-based systems introduced by Andress et al. [94] facilitate real-time image registration. This capability is indispensable in complex procedures such as percutaneous endoscopic transforaminal discectomy, where Pan et al. [95] demonstrated that visualizing the herniated disc relative to neural structures is critical for ensuring surgical success. The clinical utility of this specific system was confirmed in a prospective experiment, achieving a 95% first-time puncture success rate. Compared to traditional methods, the AI-enhanced navigation significantly reduced intraoperative radiation exposure and the number of puncture attempts, providing tangible clinical evidence of its readiness for deployment. Similarly, the AR-MISS system developed by Huang et al. [96] integrates AI and optical tracking to guide minimally invasive spine surgery. This setup enables surgeons to achieve high precision in pedicle screw placement while significantly reducing radiation exposure.

### 6.3. Human–Robot Collaborative Control

Human–robot collaboration emphasizes a synergistic relationship where the surgeon and robot operate as a unified team. In closed pelvic reduction, Pan et al. [97] proposed a Conv-BiLSTM model to recognize dynamic actions and map expert movements. This framework enables the system to learn the operational techniques of skilled surgeons and replicate them through robotic control signals.

To enhance action recognition, Pan et al. [98] subsequently upgraded this approach to a CNN-BiLSTM tri-modal fusion network. By integrating multiple data streams, the system achieved significantly higher accuracy in detecting intraoperative maneuvers, leading to more precise assistance. Beyond reactive assistance, recent research focuses on proactive interaction. A streamlined Transformer model introduced by Chen et al. [99] has further advanced the field by enabling the real-time recognition and prediction of surgical activities. This allows the robotic system to provide proactive support by anticipating the surgeon’s needs rather than merely responding to commands.

### 6.4. Intraoperative Monitoring and Feedback

Real-time feedback serves as a critical safety mechanism during surgical procedures. In robotic laminectomy, for instance, detecting the precise moment of lamina breakthrough is challenging yet vital. By analyzing electrical impedance, the system developed by Ji et al. [44] can now predict this event in real time. This capability effectively provides the surgeon with immediate intraoperative alerts to adjust their approach, rather than relying solely on late-stage visual confirmation.

### 6.5. Surgical Phase Recognition for Context-Aware Assistance

For an AI assistant to be truly effective, it must comprehend the current operative context, allowing the system to adapt its behavior to the specific surgical phase. In unicompartmental knee arthroplasty, NISHIO et al. [14] achieved this by processing footage from a wearable camera through a Conv-LSTM network with multiple CNN backbones. This setup demonstrates robust performance in clinical environments, validating wearable video as a reliable foundation for context-aware surgical support.

### 6.6. Summary: Closing the Loop from Planning to Action

Execution systems in orthopedic surgery have shifted dramatically from basic robotic controls to sophisticated AR setups and collaborative platforms. The progression is clear: the field has moved from passive navigational tools to active systems capable of executing tasks with minimal human intervention.

Historically, research focused primarily on reactive safety mechanisms, such as the robotic drills designed by Torun and Öztürk [92] to detect breakthrough and halt automatically. While effective, these early systems were relatively rigid. As technology matured, control strategies became more adaptive; for instance, Shen [91] integrated position, force, and hybrid control modes for minimally invasive knee surgery, allowing robots to adjust to intraoperative requirements in real time.

Augmented reality has transformed the visual landscape by overlaying patient anatomy and surgical instruments directly onto the operative field. This provides a significant advantage for procedures such as percutaneous endoscopic transforaminal discectomy or minimally invasive spine surgery, where Pan et al. [95], Huang et al. [96] emphasized the critical need to minimize radiation exposure while maintaining high accuracy. Nevertheless, challenges remain; maintaining perfect alignment between digital overlays and the physical world—known as real-time registration—continues to be a persistent technical challenge addressed by Andress et al. [94].

Furthermore, we are witnessing the emergence of true human–robot collaboration. Rather than acting as a mere tool, the robot is evolving into a surgical partner. By utilizing advanced architectures such as CNN-BiLSTM tri-modal fusion networks or streamlined Transformer models, Pan et al. [98], Chen et al. [99] enabled robots to detect key maneuvers and even predict subsequent surgical steps. This enables proactive assistance, though the current challenge lies in designing interfaces that ensure that these interactions remain intuitive and safe for the surgeon.

Despite this progress, significant engineering barriers persist. A primary limitation is the lack of haptic feedback; while robots are precise, they lack intrinsic tactile sensitivity. Surgeons rely on tissue resistance to modulate force, and while indirect methods like the electrical impedance monitoring used by Ji et al. [44] can warn of events such as lamina breakthrough, they do not yet provide true tactile sensation.

There is also a systemic issue regarding integration. Currently, execution systems often operate independently from perception and decision-making modules. Because these components are not fully synchronized, robots struggle to adapt to intraoperative anatomical shifts or changing surgical conditions. Addressing this requires a unified ecosystem where perception, decision, and execution are fully interconnected. Moving forward, the focus must shift from pure technical specifications toward designing user-centered systems that integrate seamlessly into the clinical workflow.

## 7. Persistent Challenges and Fundamental Limitations

Realizing the full potential of AI-assisted orthopedic surgery depends on addressing a complex network of interconnected challenges. These issues are not isolated; rather, they span technical, clinical, and systemic domains. Addressing this landscape requires a cohesive strategy that treats these sectors as a unified ecosystem. This section categorizes and summarizes these multidimensional hurdles. Persistent data limitations frequently undermine model robustness across perception, decision, and execution systems. Furthermore, significant gaps between controlled research environments and dynamic operating rooms create substantial barriers to real-world implementation. These technical hurdles, compounded by human–AI trust issues and evolving regulatory constraints, collectively hinder the development of integrated and reliable surgical AI solutions.

### 7.1. Data Dilemmas

Data dilemmas exist across all three domains of AI-assisted orthopedic surgery. The lack of high-quality, large-scale, and consistent clinical data is still a major problem. This data scarcity is more than a technical problem. It is a systemic issue that comes from the basic nature of clinical practice. Regulatory constraints and ethical considerations also contribute to the difficulty of gathering information.

In perception systems, the need for detailed anatomical annotations drives the data dilemma. As noted by Liu et al. [19], training accurate perception models requires thousands of manually annotated images, which are time-consuming and expensive to create. The inherent heterogeneity of medical images makes this problem even more difficult. These images vary significantly across different imaging modalities and scanners. And patient populations contribute to this diversity as well. Such variations lead to shifts in data distribution that degrade model performance when the system encounters new datasets or clinical sites. Hopkins et al. [67] highlighted that decision systems face similar challenges with longitudinal outcome data. Decision-making models require more than just preoperative data. They also need follow-up information regarding surgical outcomes. However, collecting this data can take months or even years. The longitudinal information is often fragmented across different healthcare systems and electronic health records, making it difficult to aggregate the data for large-scale model training. When it comes to execution systems, unique data challenges related to surgical robotics and intraoperative sensing are introduced. As discussed by Torun and Öztürk [92], models require data on surgical force, torque, and instrument interaction with tissue, which is difficult to collect in clinical settings due to privacy concerns and the complexity of data acquisition.

Beyond scarcity, bias in clinical data is another critical challenge. Most current models are trained on datasets from certain healthcare centers. These specific datasets may not represent the broader patient population. This bias will lead the model to yield suboptimal outcomes in real clinical practice. This failure is especially common for underrepresented patient groups and rare pathologies.

### 7.2. Generalization Gaps

Another fundamental challenge is the generalization gap between controlled laboratory experiments and real clinical practice. Most current models are trained and validated on clean, curated datasets in controlled settings, but their performance may degrade when applied to the noisy, dynamic environment of the operating room.

As for perception systems, the gap manifests as poor performance on images with artifacts, patient motion, or rare pathologies. For instance, the Bayesian CNN for femoral cartilage segmentation developed by Antico et al. [25] performs well on high-quality 4D ultrasound images but may struggle with images of lower quality. This gap is especially problematic in minimally invasive surgery. In these procedures, visual access to the surgical site is limited. Furthermore, the quality of the images is often compromised by the surgical environment. Furthermore, as observed by Nonnenmacher et al. [88], decision-making models trained on historical data may not adapt to changes in clinical practice as well. It is due to the lack of real-time adaptation mechanisms in current decision systems. This deficiency makes the existing limitations even worse. Currently, these systems cannot easily learn from new data during use. And they also struggle to adjust to changing clinical conditions as they remain static. In execution systems, models face the most significant generalization challenges. These systems must operate in the physical world. In this environment, patient anatomy can vary widely between individuals, and surgical conditions also change constantly during a procedure. Furthermore, instrument performance is not always consistent.

### 7.3. Human Factors

A third key issue constraining the clinical translation of AI-assisted orthopedic surgery systems lies in the human factors. Surgeons must trust these AI systems to use them effectively. They also need to understand the logic behind each recommendation. Without this trust and clarity, surgeons are reluctant to integrate AI into their standard workflows.

Interpretability is a cornerstone of trust in AI systems. As emphasized by Dasci et al. [75], transparency and explainability are essential for building confidence in AI-generated outputs. However, most current AI models in orthopedic surgery function as black boxes. They offer limited insight into their internal decision-making processes. This opacity creates a significant challenge for medical professionals. It is difficult for surgeons to know when to rely on a recommendation and when to override the system. Without transparency, the collaboration between the human and the machine remains unreliable.

Another vital aspect of the human factors is workflow integration. For example, Huang et al. [96] pointed out that many AI systems are designed as standalone tools that introduce additional steps into the surgical workflow, increasing procedural time and complexity. They disrupt established clinical routines and form a significant barrier to adoption.

### 7.4. Integration Challenges

As mentioned above, current research in AI-assisted orthopedic surgery focuses mainly on developing individual modules. These projects usually target perception, decision, or execution systems separately. They rarely focus on integrated end-to-end systems. This modular approach has driven significant progress in individual domains. But it hinders the development of truly intelligent surgical systems that can function as a whole.

The integration challenges manifest in several ways across the surgical workflow. A key challenge exists at the interface between perception and decision systems. Perception systems can quantify uncertainty in their outputs. They must communicate uncertainty to decision systems effectively. However, most decision systems are not designed to handle uncertain input data. This mismatch creates a significant problem for the overall architecture. It can lead to suboptimal decisions during the procedure and even result in unsafe execution by the surgical tools.

As for the decision-to-execution interface, the challenge is translating abstract surgical plans into concrete robotic actions. For instance, implant placement planning generates detailed strategies for the procedure, but converting these plans into robotic control signals is a complex task. This process requires precise calibration between the digital plan and the physical robot. The system must also adapt in real time to intraoperative changes. Without this constant adjustment, the robot cannot follow the surgical plan accurately in a dynamic environment. This translation gap can result in discrepancies between planned and executed actions, potentially compromising patient safety.

The lack of feedback loops between execution and perception systems is perhaps the most critical integration challenge. Most current surgical systems operate in a one-way manner. Perception informs decision-making, and these decisions then guide execution. However, execution systems generate valuable data on intraoperative conditions and surgical outcomes. Models could leverage this information to improve both perception and decision systems. The absence of this feedback limits the overall performance.

### 7.5. Regulatory and Ethical Challenges

Beyond these technical hurdles, AI-assisted orthopedic surgery faces significant regulatory and ethical challenges as well. These challenges encompass three main areas, including patient safety, data privacy, and legal liability. These issues must be addressed to achieve widespread clinical adoption.

Patient safety is the foremost regulatory concern. As emphasized by Ao et al. [69], AI-assisted surgical systems must undergo rigorous validation to ensure safety and effectiveness in clinical settings. However, traditional regulatory frameworks for medical devices are often ill-suited for AI models. This capability complicates safety assurance throughout the device lifecycle. Data privacy represents another critical challenge. LewandrowskI et al. [55] noted that AI systems require large volumes of clinical data for training, much of which contains sensitive patient information. This requirement raises significant concerns regarding data security. It also complicates compliance with strict privacy regulations. Addressing these risks calls for robust anonymization methods and secure data-sharing frameworks. Moreover, liability further complicates the adoption of AI-assisted surgical systems. As systems become increasingly autonomous, serious questions arise regarding responsibility for errors or adverse outcomes. It is unclear whether legal liability lies with the surgeon, the AI developer, or the healthcare institution. This lack of clarity can inhibit the adoption of AI by healthcare providers due to potential legal risks.

## 8. Future Directions: Toward Integrated, Adaptive, and Trustworthy Surgical AI

Building on the identified limitations of current methods, the next era of AI-assisted orthopedic surgery aims to develop integrated, adaptive, and trustworthy systems. Progress hinges on three interconnected pillars to achieve this transformation. These pillars will help researchers overcome existing barriers to clinical translation. Collectively, they guide the advancement of surgical AI from fragmented tools toward cohesive and reliable solutions.

### 8.1. Foundational Advances: Next-Generation Algorithms and Frameworks

The future of AI-assisted orthopedic surgery depends on the development of next-generation algorithms and frameworks. These new systems must address the fundamental challenges found in current methods. Three key directions mentioned below are particularly promising for this field.

#### 8.1.1. Foundation Models for Orthopedics

Foundation models represent a significant advancement for orthopedic surgery. These large-scale AI systems undergo training on diverse datasets to execute multiple tasks. This versatility gives them transformative potential in the clinical environment. A foundation model pre-trained on millions of orthopedic images, surgical videos, and clinical notes offers significant advantages. Researchers can fine-tune this model for specialized tasks like bone segmentation and surgical phase recognition. This approach directly addresses the persistent challenge of data scarcity and the need for robust feature transferability in specialized medical tasks, a strategic research direction highlighted by Devnath et al. [106] in recent systematic evaluations of medical AI systems. Such a solution is particularly useful when clinicians deal with rare pathologies or specialized surgical procedures.

Advancing foundation models for orthopedics demands collaborative efforts between AI researchers, orthopedic surgeons, and healthcare institutions. These partners must work together to curate large-scale, multi-modal datasets. They also need to design training frameworks capable of handling the complexity of orthopedic data. This cooperation will ensure that the models are both technically sound and clinically relevant.

#### 8.1.2. Causal Learning and Digital Twins

Causal learning focuses on uncovering causal relationships between variables. It does not merely identify correlations between data points. By doing so, surgical systems become more robust and generalizable. Causal models possess the capacity to reason about the effects of interventions. This ability distinguishes them from traditional correlation-based models. Such a capability is critical for surgical decision-making and preoperative planning. Digital twins provide a powerful platform for simulating surgical procedures and evaluating alternative treatment plans. These systems serve as virtual replicas of patients or surgical environments, as described by Sun et al. [107]. It complements causal learning by allowing surgeons to test various scenarios in a risk-free setting. For instance, digital twins can optimize preoperative planning and predict surgical outcomes based on individual anatomy and biomechanics.

Together, causal learning and digital twins enable personalized and precision orthopedic surgery. They allow treatment plans to be tailored to the unique anatomy of each patient. Clinicians can also account for specific pathologies and clinical profiles during the planning stage. Realizing this potential requires advances in causal inference algorithms and digital twin modeling techniques. Real-time simulation frameworks need to be developed as well.

#### 8.1.3. Uncertainty-Aware and Adaptive AI

Uncertainty-aware AI plays a pivotal role in fostering trust in orthopedic surgery systems. This technology quantifies the uncertainty behind every prediction the system makes. As a result, uncertainty quantification empowers surgeons to assess the reliability of AI-generated recommendations. Adaptive AI complements this paradigm by learning and evolving in real time. It overcomes the limitations of conventional, static models that are trained offline and remains flexible as new information becomes available during surgical procedures, an advantage noted by Chen et al. [108].

### 8.2. Toward Seamless Integration: Closing the Perception–Decision–Action Loop

The next generation of AI-assisted orthopedic surgery systems will feature seamless integration between perception, decision, and execution systems. This integration will enable the creation of closed-loop surgical assistance systems. By connecting these three components, it will move beyond simple assistance to become a truly interactive partner in the operating room.

#### 8.2.1. Collaborative Systems

Collaborative interaction among perception, decision, and execution systems represents a crucial frontier for orthopedic surgery. The three core modules can operate synergistically within this integrated approach, as noted by Ding et al. [109]. And this cooperation optimizes both surgical safety and precision during complex procedures. In traditional systems, perception feeds decision-making, and decision-making drives execution in a linear manner. However, these new systems will allow for reciprocal information flow. The structure enables dynamic adaptation to the variable demands of complex surgical scenarios. By maintaining constant feedback, the system ensures that each module informs the others throughout the entire procedure. Such synergy allows the technology to move beyond isolated tasks and toward comprehensive surgical support.

#### 8.2.2. Cognitive Robots

Cognitive robots emerge as a highly promising research direction. These systems integrate perception, decision, and execution capabilities into a unified platform and harbor the potential to transform orthopedic surgery by augmenting human cognitive capabilities, as envisioned by Schlafly et al. [110]. They perceive the surgical environment and make contextually informed decisions. The robots then execute precise surgical actions in real time. These capabilities enable autonomous or semi-autonomous procedures that enhance surgical outcomes.

Taking spinal surgery as an example, a cognitive robot uses perception modules to segment spinal anatomy and track surgical instruments accurately. It then employs decision systems to map out the optimal placement for pedicle screws. And the execution system carries out high-precision screw insertion based on that plan. Moreover, the robot can adjust its actions in real time as patient anatomy or surgical conditions change. This adaptability guarantees both safety and effectiveness during the procedure.

#### 8.2.3. Unified Learning Frameworks

Unified learning frameworks offer significant potential by optimizing entire surgical workflows rather than focusing on isolated modules. As noted by Pescio et al. [111], these frameworks learn end-to-end mappings that translate raw input data directly into surgical actions. This approach facilitates a seamless integration across the perception, decision, and execution components. By training the system as a cohesive unit, the dependencies between each stage are better managed, leading to a more fluid and responsive surgical assistant.

To achieve this, deep learning methods must be improved to advance unified learning frameworks. These methods need to process multi-modal data and capture long-term dependencies within surgical workflows. Meanwhile, these frameworks also require large-scale datasets. They have to pair raw inputs with specific surgical actions, while such resources are currently limited in the field of orthopedic surgery.

### 8.3. Human–AI Collaboration: Evolving Partnership Paradigms

Despite the promising advances in cognitive robots outlined above, the future of AI-assisted orthopedic surgery does not involve replacing surgeons with robots. Instead, researchers aim to develop collaborative systems. These systems should augment the capabilities of the surgeon and improve patient outcomes. By focusing on partnership rather than replacement, technology can better support the surgical practice.

#### 8.3.1. Context-Aware Interfaces

Context-aware interfaces adapt to the specific surgical context and the preferences of the surgeon. As demonstrated by Tu et al. [112], these systems enhance the usability and effectiveness of AI-assisted orthopedic models. They can deliver personalized support by dynamically adjusting the information they present. And they can also modify AI assistance levels based on the surgical phase and the expertise of the surgeon.

A context-aware interface for robotic spine surgery will provide comprehensive information about spinal anatomy and instrument position during the planning phase. The system will streamline the display during the execution phase to minimize distractions. And it can adjust assistance levels based on the experience of the surgeon. It will offer more guidance to trainees but allow for more autonomous operation when used by experienced surgeons.

#### 8.3.2. Shared Autonomy

Shared autonomy represents another pivotal direction for human–AI collaboration, a concept highlighted by Douglas et al. [113]. In this paradigm, surgeons and AI systems jointly control surgical procedures. This approach enables surgeons to maintain ultimate authority over the operation. At the same time, they can leverage AI to handle repetitive or technically demanding tasks. By splitting these responsibilities, the system ensures both safety and efficiency.

Realizing shared autonomy requires control algorithms that facilitate seamless transitions between human and AI control. Developers must also create interfaces that clearly communicate the system state and the level of assistance. Trust-building mechanisms are needed as well. They can help surgeons determine when they should rely on the system and when they must assume direct control.

#### 8.3.3. Continuous Learning and Personalization

Continuous learning and personalization allow AI systems to evolve through experience and provide personalized healthcare solutions, an approach advocated by Ponnambalath Mohanadas et al. [114]. In this way, the models will adapt to the specific preferences of individual surgeons. Such capabilities can significantly enhance the effectiveness of the specific models. Furthermore, these features can encourage higher adoption rates among medical professionals. When a system learns from its users, it becomes a more valuable tool in the clinical environment.

To advance this direction, researchers must prioritize improvements in online learning algorithms and model efficiency. Privacy-preserving data techniques and standardized frameworks are needed to support the development as well. Such systems must uphold patient privacy protections and adhere to all regulatory requirements.

## 9. Conclusions

This systematic review of 89 recent studies highlights a clear paradigm shift in AI-assisted orthopedic surgery: the field is rapidly moving away from isolated technical innovations toward integrated systems that encompass the entire perception–decision–execution workflow. While AI technologies have matured significantly to enhance surgical precision and personalized care, their widespread clinical adoption remains constrained by critical translation barriers, most notably data scarcity, generalization gaps across diverse clinical settings, and a lack of end-to-end system integration.

To overcome these hurdles, future research must pivot toward developing uncertainty-aware foundation models and cognitive robotic platforms characterized by bidirectional feedback loops. Ultimately, realizing the full potential of surgical AI is not about replacing human expertise, but rather forging a synergistic human–AI collaboration that seamlessly augments intraoperative performance and fundamentally improves patient outcomes.

## Figures and Tables

**Table 1:** Comparison of key characteristics across 89 studies.

Authors	Tasks	Anatomy	Modality	Dataset Size	Validation Type	Evaluation Metrics	Clinical Readiness
Kuok et al. [3]	Perception	Spine	CT	5 cases	k-fold Cross-Validation	Dice similarity coefficient	Retrospective Study
Zhang et al. [13]	Perception	Knee cartilage	Multimodal MRI	209 patients + 57 healthy volunteers	Internal Validation	Accuracy, Precision, Recall, F-measure	Retrospective Study
NISHIO et al. [14]	Perception, Execution	Knee	Video	6 videos with 218 clips	Internal Validation	Accuracy	Preclinical Study
Kadkhodamohammadi et al. [15]	Perception	Knee	Video	18 videos	6-fold cross-validation	F1-score, phase transition error	Retrospective Study
Sun et al. [16]	Perception	Cervical spine	Vibration, sound	68,000 sets	Internal Validation	Accuracy	Preclinical Study
Dunnhofer et al. [17]	Perception	Knee cartilage	Ultrasound	6 volunteers 18,278 annotated 2D slices	k-fold Cross-Validation	Dice Similarity Coefficient	Preclinical Study
Liu et al. [18]	Perception	Femoral neck femur	CT	10 cases with 2 CT scans each + 112 left and 113 right femur images (Pelvic Reference Data: https://doi.org/10.7937/TCIA.2019.WOSKQ5OO, accessed on 19 April 2026)	Random Hold-out	Dice coefficient; length error; displacement error	Retrospective Study
Liu et al. [19]	Perception, Decision	Knee femur tibia patella fibula	CT	538 patients 241,566 CT images	Random Hold-out	Dice coefficient; IOU; ASD; HD; bone resection thickness accuracy; alignment accuracy	Prospective Clinical Trial
Lu et al. [20]	Perception	Spine	CT	116 patients (VerSe2020: https://doi.org/10.17605/OSF.IO/T98FZ, accessed on 19 April 2026)	Random Hold-out	Mean Localization Error; Dice Similarity Coefficient; IoU; Pixel Accuracy; Mean Surface Distance; Hausdorff Distance; classification accuracy	Retrospective Study
Jonmohamadi et al. [21]	Perception	Knee femur ACL tibia meniscus	Arthroscopy video	3868 images	k-fold Cross-Validation	Dice similarity coefficient	Preclinical Study
Ali et al. [22]	Perception	Knee bone ACL meniscus	Arthroscopic multispectral video	not reported	k-fold Cross-Validation	Dice similarity coefficient	Preclinical Study
Ali and Pandey [23]	Perception	Knee multiple structures	Arthroscopy video	4128 phantom stereo frames + 12,695 cadaver stereo frames	Random Hold-out	L1 error; L1 gradient error; DSSIM; SSIM; Dice similarity coefficient	Preclinical Study
Antico et al. [24]	Perception	Knee femoral cartilage	Ultrasound	18,278 images	k-fold Cross-Validation	Dice similarity coefficient; Dice similarity coefficient with boundary uncertainty	Preclinical Study
Antico et al. [25]	Perception	Knee femoral cartilage	4D ultrasound	16,973 2D images	Random Hold-out	Area Under ROC Curve; Dice Similarity Coefficient; Dice Similarity Coefficient with Boundary Uncertainty	Preclinical Study
Lee et al. [26]	Perception	Spine neural tissue	Endoscopic video	2942 frames	3k-fold Cross-Validation and Random Hold-out	Dice-Sorensen coefficient; Jaccard index; precision; recall; average precision; processing time	Retrospective Study
Li et al. [27]	Perception	Kidney laparoscopy	RGB video	16 videos (EndoVis 2018 (Robotic Scene Segmentation Challenge): https://endovissub2018-roboticscenesegmentation.grand-challenge.org/Data/, accessed on 19 April 2026)	Internal Validation	CP, CR, CF1, OP, OR, OF1, HL, mAP	Preclinical Study
Zou et al. [28]	Perception	Nasopharynx	CT and MR	99 patients	Random Hold-out	precision; recall; target registration error	Retrospective Study
Yu et al. [29]	Perception	Spine	CT and X-ray	12 CT scans with X-ray pairs	not reported	mAP; matching accuracy	Retrospective Study
Fan et al. [30]	Perception	Spine	C-arm CBCT	18 patients + 4 cadavers (2016 Low Dose CT Grand Challenge: https://doi.org/10.21227/4yqw-2364, accessed on 19 April 2026)	Random Hold-out	detection rate; detection error; F1 score; intensity difference; fiducial registration error	Preclinical Study
Broessner et al. [31]	Perception	Scaphoid	US and CT	2376 US images+ 105 scaphoid models	Random Hold-out	MAE(R); MAE(t); SDE	Preclinical Study
Banach et al. [32]	Perception	Knee	Arthroscopy video	8 sequences + 1 cadaveric knee	Random Hold-out	Position Error; Rotation Error	Preclinical Study
Geng et al. [33]	Perception	Chest and pelvis	CT and X-ray	110 CT/X-ray pairs + 30 CT scans for chest + 6 patients for pelvis (Regi2D3D public dataset: https://github.com/rg2/Regi2D3D-IPCAI2020, accessed on 19 April 2026)	Random Hold-out	TRE; GFR; translation error; rotation error	Retrospective Study
Shrestha et al. [34]	Perception	Pelvis	CT and X-ray	6 CT scans + 48,600 simulated X-rays + real X-rays from 6 specimens	Random Hold-out	proj. mTRE; mTRE; GFR	Retrospective Study
Chen and Zhang [35]	Perception	Spine	CT and X-ray (simulated)	52 CT scans (Verse: https://github.com/anjany/verse, accessed on 19 April 2026) + 1000 simulated X-rays	Random Hold-out	mTRE; pose error; registration time	Retrospective Study
Ju et al. [36]	Perception	Pelvis	CT and X-ray	5000 synthetic DRRs per patient+ 10 real X-rays	Random Hold-out	MAE; SSIM; NCC	Preclinical Study
Ye et al. [37]	Perception	Head, chest, pelvis	CT and X-ray	2.25M simulated X-ray images (HaN-Seg: https://doi.org/10.1002/mp.16197, accessed on 19 April 2026), (BIMCV-COVID19+: https://github.com/BIMCV-CSUSP/BIMCV-COVID-19, accessed on 19 April 2026), (CTPelvic1k: https://github.com/MIRACLE-Center/CTPelvic1K, accessed on 19 April 2026)	Random Hold-out	MAE; mTRE; GFR	Retrospective Study
Bier et al. [38]	Perception	Pelvis	X-ray (synthetic and real)	20,000 synthetic X-rays from 20 CTs (NIH Cancer Imaging Archive: https://www.cancerimagingarchive.net/, accessed on 19 April 2026); real X-rays from 5 cadavers	Random Hold-out	mean prediction error; detection accuracy	Retrospective Study
Wang et al. [39]	Perception	Bone	Fluoroscopy	not reported	Internal Validation	Location accuracy error, Time	Preclinical Study
Lee et al. [40]	Perception, Decision	Hip	RGB video	51 participants	Random Hold-out	prediction accuracy; Gini importance; chi-square test	Retrospective Study
Viviers et al. [41]	Perception	Spine screw	X-ray and optical	18,600 images from LINEMOD (LINEMOD dataset: https://bop.felk.cvut.cz/datasets/, accessed on 19 April 2026) and custom X-ray	Random Hold-out	ADD-S; ADD; 3D translation error; 3D angle error; 2D reprojection error; inference time	Preclinical Study
Cho et al. [42]	Perception	Spine	Endoscopic video	2310 frames from 9 videos	Random Hold-out and 9-fold cross-validation	recall; precision; F1-score	Preclinical Study
Cui et al. [43]	Perception	Spine	Endoscopic video	65 patients with 22,454 images	Internal Validation	Sensitivity, Specificity, Accuracy	Retrospective Study
Ji et al. [44]	Perception, Execution	Spine lamina	Electrical impedance	246 groups 177,912 force-impedance pairs	Random Hold-out	Accuracy; time delay; breakthrough distance; Pearson correlation coefficient	Preclinical Study
Sun et al. [45]	Perception	Surgical instrument	Laparoscopic video	1850 frames (Endovis dataset: https://endovissub2017-roboticinstrumentsegmentation.grand-challenge.org/, accessed on 19 April 2026); 5000 training, 3000 test frames	Random Hold-out	PCK; classification accuracy; inference speed	Retrospective Study
Lan et al. [46]	Perception	Knee joint	A-mode ultrasound	1017 samples	not reported	classification accuracy; tracking precision (mm)	Preclinical Study
Sun et al. [47]	Perception	Bone	Acoustic microphone signal	5120 samples	Random Hold-out	Accuracy	Preclinical Study
Zhang et al. [48]	Perception	Surgical instrument	Endoscopic video	8 video sequences (EndoVis2017 dataset: https://endovissub2017-roboticinstrumentsegmentation.grand-challenge.org/, accessed on 19 April 2026)	Random Hold-out	IoU	Retrospective Study
Nwoye and Padoy [49]	Perception	Surgical tool	Laparoscopic video	20 videos+ 35,000 frames+ 65,000 boxes (CholecTrack20 dataset: https://doi.org/10.7303/syn53182642, accessed on 19 April 2026)	Random Hold-out	HOTA; DetA; LocA; AssA; MOTA; MOTP; MT; PT; ML; IDF1; IDSW; Frag; #Dets; #IDs; FPS	Retrospective Study
Xu et al. [50]	Perception	Surgical instrument	Endoscopic video	LND (1147 training, 373 test w/o occlusion, 238 test w/ occlusion)+MBF (1069 training, 209 test w/o occlusion, 387 test w/ occlusion) (SurgRIPE dataset: https://www.synapse.org/Synapse:syn51471789, accessed on 19 April 2026)	Random Hold-out	ADD; Avg Acc; Translation Error; Rotation Error; proj2d; mmd5	Retrospective Study
Yang et al. [51]	Perception	Surgical instrument	Endoscopic video	8 video (SurgRIPE dataset: https://www.synapse.org/Synapse:syn51471789, accessed on 19 April 2026) + 590 images (kvasir-instrument dataset: https://doi.org/10.17605/OSF.IO/KP6MY, accessed on 19 April 2026)	Random Hold-out and 5-fold cross-validation	Dice; mIOU	Retrospective Study
Sheng et al. [52]	Perception	Surgical instruments	Endoscopic video	8k images (CholecSeg8k: https://www.kaggle.com/datasets/newslab/cholecseg8k, accessed on 19 April 2026) + 20 videos	Internal Validation	mIoU	Preclinical Study
Jonmohamadi et al. [53]	Perception	Knee femur ACL meniscus	Arthroscopy video	38,500 frames + 12,000 frames	External Validation	ATE; photometric reprojection loss	Preclinical Study
Abel et al. [54]	Perception, Decision	Lumbar spine	MRI and CT	16 patients	Internal Validation	Lateral deviation; axial angle; sagittal angle; vertebral length; vertebral width; pedicle height; pedicle width; ICC	Retrospective Study
LewandrowskI et al. [55]	Perception	Lumbar spine	MRI	3560 patients 17,800 disc levels	Random Hold-out	accuracy; sensitivity; specificity	Retrospective Study
Fang and Wang [56]	Perception	Knee cartilage	MRI	1043 images	Random Hold-out	SEN; SPE; PPV; NPV; FPR; BER; ACC; AUC	Retrospective Study
Ghauri et al. [57]	Perception	Spine	X-ray	967 images	Random Hold-out	accuracy; precision; recall; sensitivity; specificity; AUC	Retrospective Study
Xiang et al. [58]	Perception	Lumbar spine nucleus pulposus	Ultrasound	152 samples	Random Hold-out	Accuracy; Precision; Sensitivity; Specificity; F1	Retrospective Study
Voinea et al. [59]	Perception	Knee joint	MRI	1556 exams	Random Hold-out; Monte-Carlo cross-validation; k-fold Cross-Validation	accuracy; precision; recall; F1; ROC-AUC	Retrospective Study
Burlison et al. [60]	Perception	Knee femur	CT and MR	424 image pairs	External Validation	Pearson’s r; CCC; R-squared; bias; limits of agreement	Retrospective Study
Antico et al. [61]	Perception	Knee femoral cartilage	Ultrasound	38,656 images	5k-fold Cross-Validation	accuracy; specificity; sensitivity; AUC; percent agreement; Cohen’s kappa	Preclinical Study
Wang et al. [62]	Perception	Surgical scene	Stereo endoscopic video	1024 image pairs (Flickr1024: https://yingqianwang.github.io/Flickr1024, accessed on 19 April 2026) for training; 150 test pairs (EndoVis2017 dataset: https://endovissub2017-roboticinstrumentsegmentation.grand-challenge.org/, accessed on 19 April 2026)	External Validation	PSNR; SSIM	Preclinical Study
Gunaratne et al. [63]	Perception	Human joint tissue	Diffuse reflectance and auto-fluorescence spectroscopy	3060 spectra	k-fold Cross-Validation	accuracy	Preclinical Study
Gunaratne et al. [64]	Perception	Knee joint	Diffuse reflectance spectroscopy	3043 spectra	k-fold Cross-Validation	accuracy; sensitivity	Preclinical Study
Cui et al. [65]	Perception	Spine nerve and dura mater	Endoscopic video	4829 images	Random Hold-out	accuracy; sensitivity; specificity	Retrospective Study
Yao et al. [66]	Perception	Spine spinal cord nucleus pulposus adipose tissue nerve root	Ultrasound	758 samples	Random Hold-out	Accuracy; Precision; Sensitivity; Specificity; F1; Latency	Preclinical Study
Hopkins et al. [67]	Decision	Spine	Clinical data	4046 patients	k-fold Cross-Validation	AUC; sensitivity; specificity; PPV; NPV	Retrospective Study
Zhang et al. [4]	Decision	Spine	CT	not reported	Internal Validation	not reported	Preclinical Study
Cui [68]	Decision	Spine	not reported	not reported	Internal Validation	Accuracy, time, success rate, adaptability score	Preclinical Study
Ao et al. [69]	Decision	Spine pedicle screw	CT, ultrasound, surface reconstruction	5 human models+5 water phantoms+ 35 real vertebrae	External Validation	Safe rate; insertion ratio; breach rate; Gertzbein-Robbins classification; direction error; trajectory distance	Preclinical Study
Fauser et al. [70]	Decision	Temporal bone	CT	24 patients	2k-fold Cross-Validation	Dice coefficient; sensitivity; planning success rate	Retrospective Study
Siemionow et al. [71]	Decision	Lumbar spine pedicle	CT	20 patients	not reported	Zidichavsky Score; Ravi Grade; Gertzbein Grade	Retrospective Study
Wang et al. [72]	Decision	Pelvis	CT	481 annotated CT scans for training+ 12 patients for testing	External Validation	Surgical time; VAS; ECOG; screw placement accuracy	Prospective Clinical Trial
Campagner et al. [73]	Decision	Spine lumbar	Biomarkers and clinical data	72 patients	External Validation	accuracy; AUC; F1 score	Retrospective Study
Sánchez-Guillén et al. [74]	Decision	Musculoskeletal system	Survey data	651 surgeons	Internal Validation	accuracy; sensitivity; specificity; predictive values	Retrospective Study
Dasci et al. [75]	Decision	Hip	ChatGPT-4o and Google Search	40 FAQs	not reported	response accuracy score; Rothwell classification; source categorization; Cohen’s kappa	Retrospective Study
Pasha and Flynn [76]	Decision	Spine	X-ray	67 AIS patients	Internal Validation	silhouette value; classification accuracy; likelihood ratio	Retrospective Study
Karhade et al. [77]	Decision	Spine	Clinical and laboratory variables	4304 patients	External Validation	AUC, Brier score, Calibration, Decision-curve analysis	Retrospective Study
Toyoda et al. [78]	Decision	Lumbar spine	Clinical and radiographic data	331 patients	10k-fold Cross-Validation	accuracy; sensitivity; specificity; PPV; NPV	Retrospective Study
Chen et al. [79]	Decision	L5-S1 lumbar disc	MRI CT X-ray clinical data	309 patients (https://doi.org/10.5281/zenodo.15565334, accessed on 19 April 2026)	Random Hold-out	accuracy; sensitivity; specificity; PPV; NPV; F1; AUC	Retrospective Study
Zhang et al. [80]	Decision	Knee	Musculoskeletal multibody dynamics simulation data	343 sets	5k-fold Cross-Validation	RMSE; Pearson correlation coefficient	Retrospective Study
Ahammad et al. [81]	Decision	Spine	Sensor data	310 cases (https://archive.ics.uci.edu/dataset/212/vertebral-column, accessed on 19 April 2026)	Internal Validation	TP rate; accuracy; error rate; precision; recall; standard error	Retrospective Study
Liao et al. [82]	Decision	Spine	Clinical data	1136 patients	Internal Validation	AUC; accuracy; RMSE	Retrospective Study
Cavazos et al. [83]	Decision	Knee	Clinical data	2093 patients	Random Hold-out	AUC; accuracy; sensitivity; specificity	Retrospective Study
Yamada et al. [84]	Decision	Lumbosacral spine L5-S1 L5-L6	MRI and CT	52 patients	Internal Validation	JOA score; VAS	Retrospective Study
Liu et al. [85]	Decision	Pelvis	CT	40 public CT + 10 patients (PelvisAtlas dataset: https://github.com/I-STAR/PelvisAtlas, accessed on 19 April 2026)	Internal Validation	Reconstruction error, Force prediction error, Modeling time, Pearson correlation, Non-parametric test	Retrospective Study
Thibeault et al. [86]	Decision	Spine	X-ray & 3D model	816 samples	random hold-out	3D RMS, IoU, Chamfer distance, Cobb angle difference, lordosis angle difference	Retrospective Study
Kim et al. [87]	Decision	Elbow joint	IMU sensor	180 measurements	Internal Validation	Precision, Recall, F1-Score, classification accuracy	Retrospective Study
Nonnenmacher et al. [88]	Decision	Hip	Clinical data	235 patients	Internal Validation	AIC, *p*-value	Retrospective Study
Subramanian et al. [89]	Decision	Spine	Text	not reported	not reported	not reported	Simulated Deployment
Elmakias and Dabran [90]	Decision	Neck, wrist	VR sensor	176 recordings	Internal Validation	Accuracy	Preclinical Study
Shen [91]	Execution	Knee joint	CT	not reported	not reported	not reported	Preclinical Study
Torun and Öztürk [92]	Execution	Bone	Closed-loop sensor signals	90 holes	5-fold cross-validation	Overall accuracy, Breakthrough accuracy, CPU time	Preclinical Study
Li et al. [93]	Execution	Spine	Force, position, optical	1054 samples	Internal Validation	Accuracy, recognition rate	Preclinical Study
Andress et al. [94]	Execution	Femur/Pelvis	Fluoroscopy, RGB, AR	not reported	Internal Validation	Positional error, rotational error, RMSE, precision, distance error, procedure time, X-ray count	Preclinical Study
Pan et al. [95]	Execution	Spine	Fluoroscopy, optical tracking, depth camera	20 patients + 10 dogs	Prospective Validation	Positioning error, Orientation error, Fluoroscopy times, VAS, ODI	Prospective Clinical Trial
Huang et al. [96]	Execution	Spine	AR, optical tracking, fluoroscopy	20 dogs	Internal Validation	Distance error, Angle error	Preclinical Study
Pan et al. [97]	Execution	Pelvis	Wearable sensors	20 subjects + 1000 action sets	5-fold cross-validation	Accuracy, Precision, Recall, F1-Score	Preclinical Study
Pan et al. [98]	Execution	Pelvis	Wearable sensors	6804 action segments	5-fold cross-validation	Accuracy, F1-Score	Preclinical Study
Chen et al. [99]	Execution	Surgical robotics	Kinematic data	8 subjects+ 103 trials	LOUO cross-validation	Accuracy, RMSE, MAE, MAPE, execution time	Preclinical Study

## Data Availability

No datasets were generated or analyzed during the current study.
